# Prevalence and progression of rheumatic heart disease: a global systematic review and meta-analysis of population-based echocardiographic studies

**DOI:** 10.1038/s41598-019-53540-4

**Published:** 2019-11-19

**Authors:** Jean Jacques Noubiap, Valirie N. Agbor, Jean Joel Bigna, Arnaud D. Kaze, Ulrich Flore Nyaga, Bongani M. Mayosi

**Affiliations:** 1 Centre for Heart Rhythm Disorders, University of Adelaide and Royal Adelaide Hospital, Adelaide, Australia; 2 Nuffield Department of Population Health, University of Oxford, Oxford, United Kingdom; 3 Department of Epidemiology and Public Health, Centre Pasteur of Cameroon, Yaoundé, Cameroon; 4 School of Public Health, Faculty of Medicine, University of Paris Sud XI, Le Kremlin-Bicêtre, France; 5 Department of Medicine, Brigham and Women’s Hospital, Harvard Medical School, Boston, MA USA; 6 Department of Medicine, University of Maryland Medical Center Midtown Campus, Baltimore, MD USA; 7 Department of Internal Medicine and Specialties, Faculty of Medicine and Biomedical Sciences, University of Yaoundé I, Yaoundé, Cameroon; 8 The Dean’s Office, Faculty of Health Sciences, University of Cape Town, Cape Town, South Africa

**Keywords:** Cardiology, Valvular disease, Epidemiology

## Abstract

This systematic review and meta-analysis aimed to provide a contemporaneous estimate of the global burden of rheumatic heart disease (RHD) from echocardiographic population-based studies. We searched multiple databases between January 01, 1996 and October 17, 2017. Random-effect meta-analysis was used to pool data. We included 82 studies (1,090,792 participant) reporting data on the prevalence of RHD and 9 studies on the evolution of RHD lesions. The pooled prevalence of RHD was 26.1‰ (95%CI 19.2–33.1) and 11.3‰ (95%CI 7.2–16.2) for studies which used the World Heart Federation (WHF) and World Health Organization (WHO) criteria, respectively. The prevalence of RHD varied inversely with the level of a country’s income, was lower with the WHO criteria compared to the WHF criteria, and was lowest in South East Asia. Definite RHD progressed in 7.5% (95% CI 1.5–17.6) of the cases, while 60.7% (95% CI 42.4–77.5) of cases remained stable over the course of follow-up. The proportion of cases borderline RHD who progressed to definite RHD was 11.3% (95% CI 6.9–16.5). The prevalence of RHD across WHO regions remains high. The highest prevalence of RHD was noted among studies which used the WHF diagnostic criteria. Definite RHD tends to progress or remain stable over time.

## Introduction

Rheumatic heart disease (RHD), an inflammatory heart valve condition, is a chronic sequel of acute rheumatic fever (ARF). ARF is a multisystem disease resulting from an autoimmune reaction to group A streptococcal (GAS) pharyngitis in genetically susceptible individuals^1^. RHD causes inflammation of the cardiac valves, initially leading to clinically silent valvular disease and ultimately severe permanent damage. Individuals with RHD are at increased risk of complications such as congestive heart failure, arrhythmias including atrial fibrillation, stroke, infective endocarditis, poor maternal and fetal outcomes, and premature death^2–4^. RHD is the most common cause of acquired heart disease in children and young adults globally^5^.

ARF and RHD are diseases of poverty, driven by poor sanitation, overcrowding, malnutrition and limited access to health care. In 2015, RHD affected 33.4 million people globally, and caused 319,400 deaths, nearly all of which occurred in LMIC^5^. This heavy burden of RHD contrasts with persistent neglect of the condition on national health agendas in endemic countries^6^.

To tackle the burden of RHD, the World Heart Federation (WHF) released in 2013 a position statement on the prevention and control of RHD, with the ambitious goal of achieving a 25% reduction in premature deaths from ARF and RHD among individuals aged <25 years by 2025^7^. To achieve this goal, comprehensive and effective national disease programs should be developed and integrated into existing efforts by ministries of health. A major impediment is the lack of “true” burden of disease estimates on local, national, and international levels which can be used for the implementation of existing evidence-based, cost-effective approaches to preventing GAS/ARF and treating RHD^7^.

Echocardiography is the most cost-effective tool for population screening and estimating the prevalence of RHD^8^. About five years ago, Rothenbühler *et al*. published a meta-analysis of the prevalence of RHD among children and adolescents, highlighting the substantial burden of disease in endemic regions^9^. The major flaw of this meta-analysis was to pool together estimates from studies which used various echocardiographic screening approaches and criteria. Additionally, a large number of echocardiographic screening studies have been published subsequent to the publication of this study. We present herein an updated systematic review and meta-analysis population-based echocardiographic studies, to estimate the prevalence of RHD according to diagnostic protocol and criteria, and to assess the evolution of clinically silent RHD.

## Methods

### Design and registration

This systematic review and meta-analysis is reported in accordance with the Preferred Reporting Items for Systematic Reviews and Meta-analyses (PRISMA)^10^. The protocol was registered in PROSPERO (registration number CRD42017068732).

### Search strategy for identifying relevant studies

We performed a comprehensive and exhaustive search of PubMed/MEDLINE, Excerpta Medica Database (EMBASE), African Journals Online (AJOL), the Latin-American and Caribbean System and the Cochrane Database of Systematic Reviews to identify all relevant population-based studies estimating the prevalence of RHD using echocardiography and published between January 01, 1996 and October 17, 2017. We conceived and applied a search strategy based on the combination of terms related to RHD, ARF and echocardiography. The search strategies for all the databases are available in the Appendix (Supplementary Tables 1–5). To supplement these bibliographic database searches, we also scrutinized references of all relevant research articles and reviews to identify additional potential data sources.

### Criteria for eligibility

To be included in this systematic review, studies had to be population-based (i.e., school-based or community-based) with a sample size of at least 300 participants, and reporting on the prevalence of RHD detected or confirmed using echocardiography, irrespective of the age of participants. We excluded studies which used only auscultation to screen for RHD with no confirmatory echocardiography, and those reporting primarily on ARF or GAS infections.

### Selection of studies for inclusion in the review

Two review authors (JJN and ADK) independently screened the titles and abstracts of articles retrieved from literature search, and the full-texts of articles found potentially eligible were obtained and further assessed for final inclusion. For studies published in more than one report (duplicates), the most comprehensive report of the largest sample size was considered. Disagreements were resolved through discussions between investigators until a consensus was reached.

### Appraisal of the methodological quality of included studies

The methodological quality of included studies was assessed using an adapted version of the tool developed by Hoy and colleagues to evaluate the risk of bias in prevalence studies^11^. Each item was assigned a score of 1 (yes) or 0 (no), and scores were summed across items to generate an overall quality score that ranged from 0 to 9. Studies at low risk of bias had scores of 7 or higher, moderate a score of 4–6, and high a score of 3 or lower. Two review authors (VNA and UFN) independently assessed study quality; disagreements were resolved by consensus.

### Data extraction and management

Two review authors (VNA and UFN) independently extracted relevant data from included studies using a preconceived and standardized abstraction form. Disagreements between these authors were reconciled through discussion and consensus. Two review authors (JJN and JJB) cross-checked the database for errors. Data were extracted from each study on: the surname of the first author, year of publication, area (rural vs urban), country of recruitment of participants, study design, sampling method, male proportion, age distribution, setting (school-based vs community-based), diagnostic approach (auscultation only for screening with second-line echocardiographic confirmation [auscultation > echo], first-line echocardiography with or without auscultation with second line echocardiographic confirmation [echo > echo], and first line echocardiography without confirmation [echo > nothing]), diagnostic criteria (World Health Organization, WHF criteria, and others), the number of participants with clinically silent and manifest RHD, and with borderline and definite RHD. Clinically manifest RHD was defined as the presence of a heart murmur on cardiac auscultation with evidence of RHD on echocardiography (pathological mitral regurgitation or stenosis and/or morphological features of RHD), while absence of heart murmur with echocardiographic evidence of RHD were considered as cases of clinically silent RHD^7^. Where relevant data were not available, we contacted the corresponding author to request the information. Using the country in which the study was conducted in and year of recruitment, we assigned gross domestic product per capita (GDP) in United State dollars^12^, WHO regions^13^, United Nations Statistics Division (UNSD) of countries by continent^14^, situation or not in endemic area^15^, human development index (HDI)^16^, the 2016 level of income^17^, and GINI coefficient^18^. For multinational studies, data were presented according to the country where the study was conducted in.

### Data synthesis and analysis

All analyses were performed using ‘*meta*’ packages of R (version 3.3.3) (The R foundation for statistical computing, Vienna, Austria). Freeman-Tukey double arcsine transformation was used to pool data by random effect meta-analysis^19^. Following crude overall prevalence, a sensitivity analysis was conducted considering only studies with a low risk of bias. We assessed inter-rater agreement for inclusion and quality assessment using Cohen’s kappa (κ) coefficient. All prevalence estimates were reported per 1000 people (‰) with their 95% confidence interval (95%CI) and their 95% predictive interval (95%PI).

We appraised heterogeneity between studies using Cochran’s Q statistic, H and the I^2^ statistics^20,21^, which estimate the percentage of total variation across studies due to true between-study difference rather than chance, with I^2^ values of 25%, 50% and 75% representing low, medium and substantial heterogeneity, respectively. We explored sources of heterogeneity through subgroup and meta-regression analyses defined by mean/median age, proportion of males, diagnostic approach, diagnostic criteria, study setting, area, WHO regions, UNSD of countries, clinical significance of RHD, level of income, GINI, HDI, GDP, and situation in endemic area. Comparisons between subgroups were performed using the Q-test based on the Analysis of the Variance (ANOVA). Univariable and multivariable meta-analyses were used. To be included in the multivariable analysis, a p value < 0.20 was required in univariable analysis. For categorical variables, the global p value was considered for inclusion in the multivariable model. We applied a manual backward selection procedure to identify factors independently associated with the variation of overall prevalence of RHD. We successively removed variables from the model if p value > 0.10. In the case of non-linear distribution, we log-transformed the covariate before conducting the meta-regression analyses. Publication bias was evaluated with funnel plots supplemented by formal statistical assessment using Egger’s test^22^. A p value < 0.10 was considered statistically significant to detect publication bias.

## Results

### The review process

**Figure 1:**
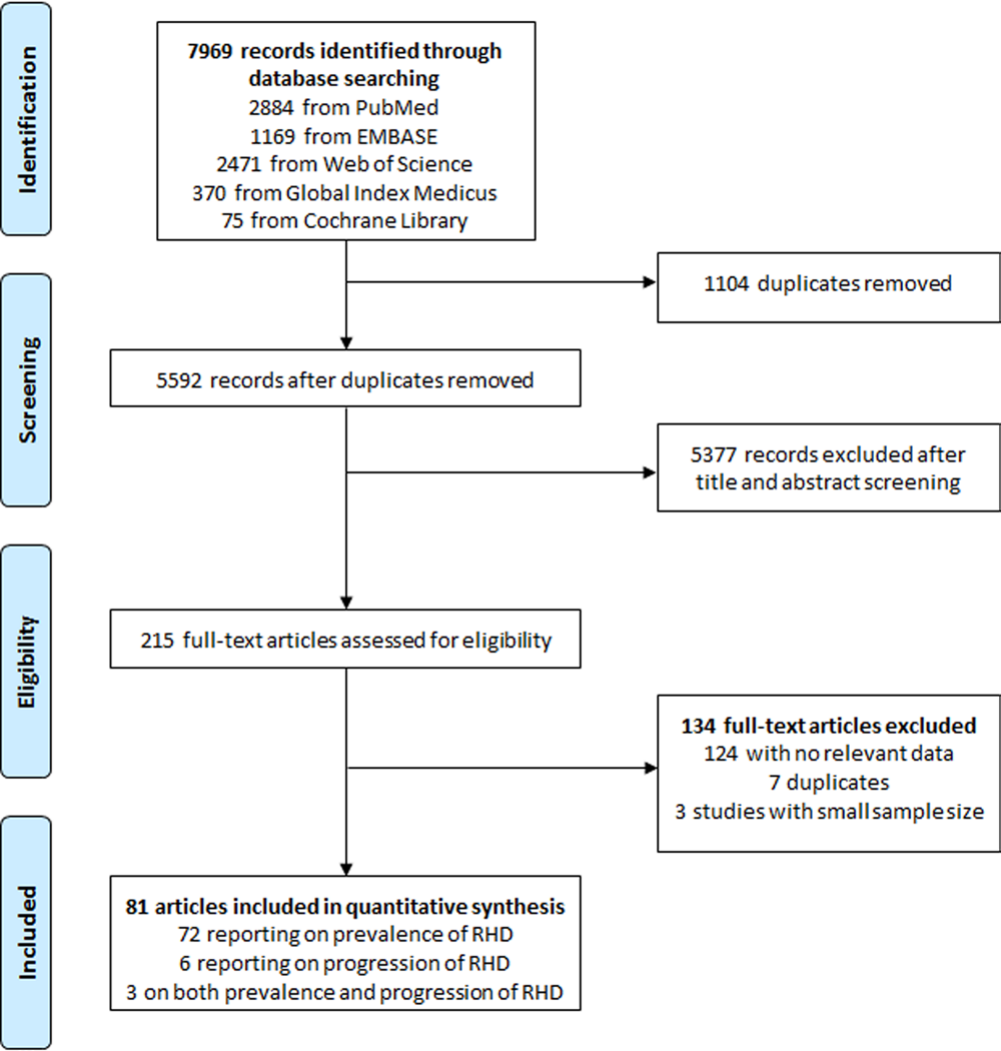
Flow diagram for selection of articles for inclusion in the meta-analysis.

A total of 7969 records were retrieved via databases searches. After removing duplicates, we screened the titles and abstracts of 5592 records, of which 215 were selected for full-text review. Of these, 81 articles were included in the review, 72 providing data on the prevalence of RHD (in a total of 82 individual studies), 6 reporting data on the evolution of RHD lesions, and 3 on both the prevalence and the evolution of RHD (Fig. 1), all published from 1996 to 2017^23–103^. Inter-rater agreements for inclusion based on titles and abstracts, full texts, and for assessment of the methodological quality of finally included studies between review authors were κ = 0.68, 0.98, and 0.87 respectively.

### Characteristics of included studies reporting on the prevalence of RHD

Regarding methodological quality, 51 (62%) had low risk of bias, 30 (37%) had moderate risk of bias, and one (1%) had high risk of bias. The inter-rater agreement for quality assessment was excellent (κ = 0.97). The characteristics of included studies from 35 countries are summarized in the Appendix (Supplementary Tables 6–8). Most of studies were school-based (78%, n = 64), conducted both in urban and rural settings, in endemic areas (84.1%, n = 69) of low-middle income countries (41.5%, n = 34). The most represented WHO regions were Africa (26.8%, n = 22), South-East Asia (26.8%, n = 22) and Western Pacific (24.4%, n = 20). On the other hand, the most represented UNSD continent were Asia and Africa. The majority of studies used the WHF (39%, n = 32) and WHO criteria (36.6%, n = 30), and the echo > echo procedure (51.1%, n = 46).

Participants were recruited from 1991 to 2015. The mean or median age of participants varied from 8 to 48 years (48 studies); range from 3 to 74 years (74 studies). The proportion of male participants varied from 30% to 65% (58 studies). The sample size in included studies varied from 337 to 229,829 participants. The median GDP, HDI and GINI coefficient were 1346 USD (range: 140–40911), 0.624 (range: 0.418–0.939; 79 studies) and 0.36 (range: 0.31–0.63; 76 studies), respectively.

### Overall prevalence of rheumatic heart disease

**Figure 2:**
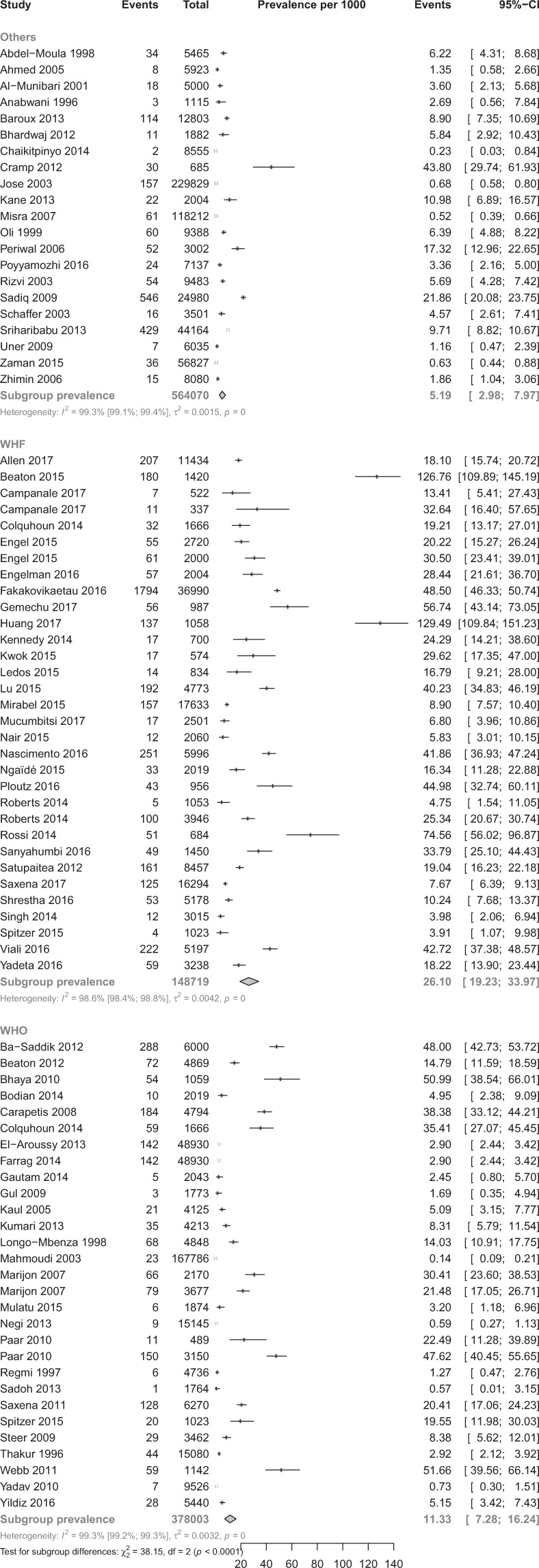
Prevalence of overall cases of rheumatic heart disease across studies according to diagnostic criteria.

**Figure 3:**
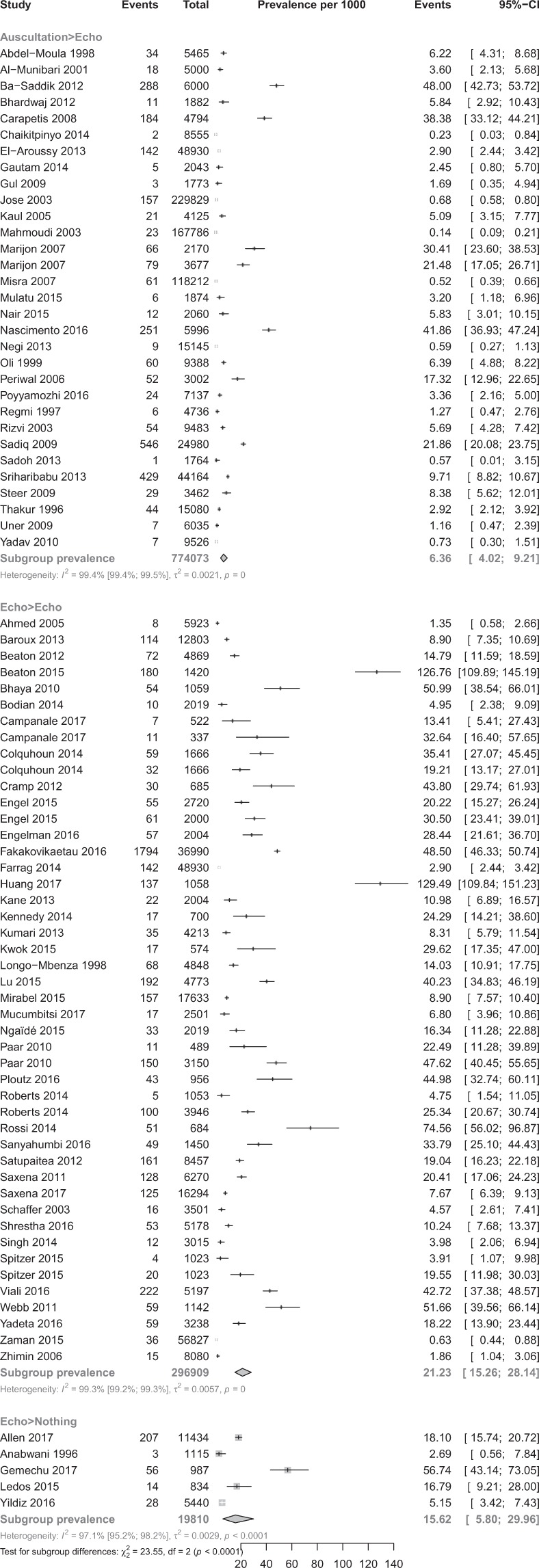
Prevalence of overall cases of rheumatic heart disease across studies according to screening strategy.

Table 1 shows the pooled prevalence of RHD in the included studies. In a total sample of 1,090,792 participants from 82 studies, the overall RHD prevalence estimates were 26.1‰ (95%CI 19.2–33.1), 11.3‰ (9%CI 7.2–16.2), and 5.2‰ (95%CI 3.0–8.0) with WHF, WHO and other criteria respectively (p < 0.0001) (Fig. 2). The overall RHD prevalence estimates were 6.4‰ (95%CI 4.0–9.2) and 21.2‰ (95%CI 15.3–28.1) with auscultation > echo and echo > echo procedures, respectively (p < 0.0001) (Fig. 3). Prevalence estimates of sensitivity analysis including only studies with low risk bias were in the range of the crude analysis. There was a wide variation of the range of the 95% PI.

**Table 1:** Summary statistics from meta-analyses of prevalence studies on rheumatic heart disease.

RHD definition	Criteria details	N Studies	N Participants	Prevalence, per 1000 (95%CI)	Prediction interval	I² (95%CI)	H (95%CI)	p heterogeneity	p Egger test	p difference criteria
**Overall**										
	**Criteria**									
	- WHF	32	148719	26.1 (19.2–33.1)	0.5–86.0	98.6 (98.4–98.8)	8.6 (8.0–9.2)	< 0.0001	0.961	< 0.0001
	- WHO	39	378003	11.3 (7.2–16.2)	0.0–50.1	99.3 (99.2–99.3)	11.4 (10.7–12.1)	< 0.0001	< 0.0001	
	- Others	21	564070	5.2 (3.0–8.0)	0.0–24.4	99.3 (99.1–99.4)	11.6 (10.8–12.5)	< 0.0001	0.021	
	**Diagnostic procedure**									
	- A > E	31	774073	6.4 (4.0–9.2)	0.0–30.0	99.4 (99.4–99.5)	13.3 (12.6–14.0)	< 0.0001	0.0009	< 0.0001
	- E > E	46	296909	21.2 (15.3–28.1)	0.0–88.0	99.3 (99.2–99.3)	11.8 (11.2–12.3)	< 0.0001	0.006	
	- E > N	5	19810	15.6 (5.8–30.0)	0.0–95.2	97.1 (95.2–98.2)	5.8 (4.6–7.5)	< 0.0001	0.898	
**Overall, low risk of bias studies**										
	**Criteria**									
	- WHF	21	116602	30.6 (20.8–42.2)	0.3–104.0	99.0 (98.8–99.1)	10.0 (9.2–10.8)	< 0.0001	0.787	0.0008
	- WHO	21	349486	11.7 (7.1–17.4)	0.0–50.2	99.3 (99.2–99.4)	12.3 (11.5–13.2)	< 0.0001	< 0.0001	
	- Others	9	186116	9.2 (3.0–18.7)	0.0–61.8	99.5 (99.4–99.6)	14.3 (12.9–15.7)	< 0.0001	0.092	
	**Diagnostic procedure**									
	- A > E	16	436227	9.3 (4.7–15.5)	0.0–48.3	99.6 (99.6–99.7)	16.8 (15.7–17.9)	< 0.0001	0.003	0.0014
	- E > E	31	197282	23.1 (15.7–31.8)	0.0–92.2	99.2 (99.1–99.3)	11.4 (10.7–12.1)	< 0.0001	0.177	
	- E > N	4	18695	20.3 (7.8–12.8)	0.0–153.5	97.5 (95.6–98.5)	6.3 (4.8–8.2)	< 0.0001	0.668	
**Definite**										
	**Criteria**									
	- WHF	32	148719	11.4 (6.7–17.2)	0.0–61.6	98.8 (98.6–98.9)	9.1 (8.5–9.7)	< 0.0001	0.083	0.008
	- WHO	29	378003	6.4 (3.9–9.6)	0.0–30.7	98.9 (98.7–99.0)	9.4 (8.8–10.1)	< 0.0001	< 0.0001	
	- Others	21	564070	3.7 (1.9–6.0)	0.0–19.4	99.2 (99.0–99.3)	11.0 (10.2–11.8)	< 0.0001	0.054	
	**Diagnostic procedure**									
	- A > E	31	774073	4.6 (2.8–6.8)	0.0–21.8	99.2 (99.2–99.3)	11.5 (10.9–12.2)	< 0.0001	0.003	0.031
	- E > E	46	296909	9.3 (5.7–13.7)	0.0–56.1	99.1 (99.1–99.2)	10.8 (10.3–11.3)	< 0.0001	0.291	
	- E > N	5	19810	9.7 (4.3–17.2)	0.0–48.7	93.4 (87.5–96.5)	3.9 (2.8–5.3)	< 0.0001	0.822	
**Borderline/Probable**										
	**Criteria**									
	- WHF (Borderline)	28	93059	15.2 (10.5–20.7)	0.0–37.8	97.4 (96.8–97.8)	6.2 (5.6–6.8)	< 0.0001	0.278	0.0004
	- WHO (Probable)	10	56331	5.6 (2.3–10.4)	0.9–12.5	97.3 (96.3–98.1)	6.1 (5.2–7.2)	< 0.0001	0.011	
	- Others (Probable)	2	2689	5.9 (1.0–14.5)	NA	74.2 (0.0–94.2)	2.0	0.049	NA	
	**Diagnostic procedure**									
	- A > E	7	52537	5.8 (0.8–15.3)	0.0–61.9	99.1 (98.8–99.3)	10.6 (9.3–12.2)	< 0.0001	0.233	0.279
	- E > E	30	86287	13.7 (9.5–18.6)	0.0–51.2	96.9 (96.2–97.4)	5.6 (5.1–6.2)	< 0.0001	0.024	
	- E > N	3	13255	11.7 (5.8–19.6)	0.0–216.2	79.8 (36.1–93.6)	2.2 (1.2–4.0)	0.007	0.345	
**Clinically Manifest**										
	**Criteria**									
	- WHF	10	67933	4.7 (1.9–8.8)	0.0–24.9	96.1 (94.5–97.3)	5.1 (4.2–6.1)	< 0.0001	0.613	0.100
	- WHO	18	124940	1.7 (1.0–2.6)	0.0–6.2	86.2 (79.6–90.6)	2.7 (2.2–3.3)	< 0.0001	0.006	
	- Others	6	21396	2.6 (0.1–7.7)	0.0–33.6	96.0 (93.4–97.5)	5.0 (3.9–6.4)	< 0.0001	0.402	
	**Diagnostic procedure**									
	- A > E	12	72912	1.9 (0.7–3.5)	0.0–11.0	94.0 (91.2–95.9)	4.1 (3.8–4.9)	< 0.0001	0.103	0.303
	- E > E	20	139408	3.3 (1.8–5.1)	0.0–14.4	94.8 (93.2–96.1)	4.4 (3.8–5.0)	< 0.0001	0.150	
	- E > N	2	1949	2.0 (0.3–4.7)	0.3–4.7	0.0	1.0	0.538	NA	
**Clinically Silent**										
	**Criteria**									
	- WHF	9	67099	11.6 (5.2–17.8)	0.0–93.6	99.3 (99.1–99.4)	11.8 (10.5–13.2)	< 0.0001	0.1548	0.302
	- WHO	16	74246	10.6 (5.2–17.8)	0.0–55.8	98.5 (98.1–98.8)	8.1 (7.3–9.0)	< 0.0001	0.004	
	- Others	4	14246	3.8 (0.1–11.8)	3.5–6.7	95.8 (92.0–97.8)	4.9 (3.5–6.7)	< 0.0001	0.098	
	**Diagnostic procedure**									
	- A > E	10	65113	2.6 (0.6–6.0)	0.0–22.6	97.6 (96.7–98.2)	6.4 (5.5–7.5)	< 0.0001	0.113	0.0003
	- E > E	19	90478	15.5 (8.7–24.2)	0.0–71.7	98.6 (98.3–98.8)	8.5 (7.7–9.3)	< 0.0001	0.108	
	- E > N	0	0	NA	NA	NA	NA	NA	NA	

NA: not applicable; WHF: World Heart Federation; WHO: World Heart Organization; A > E = Auscultation only for screening followed with echography confirmation; E > E: Echography +/− auscultation for screening followed with echography for confirmation; E > N: Echography only for screening without echography confirmation; CI = confidence interval.

Definite RHD prevalence varied from 3.7‰ to 11.4‰ depending on the diagnostic criteria or procedure used with significant difference between diagnostic criteria and procedure. Borderline or probable RHD prevalence varied from 5.6‰ to 15.2‰ with difference between diagnostic criteria and without difference between diagnostic procedures. Clinically manifest RHD prevalence varied from 1.7‰ to 2.6‰ without difference between diagnostic criteria and procedure. Clinically silent RHD prevalence varied from 2.6‰ to 15.5‰ with difference between diagnostic procedures but not between diagnostic criteria (Table 1).

### Subgroup analyses

When considering WHF and WHO criteria, the prevalence differed between levels of income but this difference was not found when considering diagnostic procedure. Regardless of diagnostic criteria or procedure, there was no difference between endemic and non-endemic areas, between rural and urban areas, and between community-based and school-based studies. Regardless of diagnostic criteria or procedure, there was difference between UNSD regions and WHO regions (Appendix, Supplementary Table 9).

### Factors associated with the prevalence of rheumatic heart disease

In the univariable meta-regression analysis, the RHD prevalence was associated with the year of publication, GDP, GINI coefficient, proportion of males, diagnostic procedure, diagnostic criteria, WHO regions, UNSD of countries, and level of income. In the final multivariable model, the prevalence of RHD was lowest in South-East Asia compared to WHO regions. The RHD significantly decreased with rising level of income; low income countries had higher RHD prevalence. Compared with the WHF diagnostic criteria, the prevalence of RHD estimated with WHO criteria was significantly lower. UNSD of countries by continent was not associated with RHD prevalence. Variables included in the final model explained 57.3% of the 98.4% residual heterogeneity of the RHD prevalence (Table 2).

**Table 2:** Factors associated with the variation of the global prevalence of rheumatic heart disease.

Variables (reference)	Univariable model		Multivariable final model^†^	
	P value	Coefficient %, (95% CI)	P value	Adjusted coefficient (95%CI)
**Year of publication**	0.0071	0.0049 (0.0028; 0.0071)	*	
**Endemic area**	0.9561	0.0011 (−0.0376; 0.0398)		
**Gross national income per capita**	0.0358	0.0103 (0.0007; 0.0199)	*	
**GINI**	0.0114	0.0032 (0.0007; 0.0056)	*	
**HDI**	0.8740	0.0093 (−0.1061; 0.1248)		
**%Male**	<0.0001	−0.0043 (−0.0063; −0.0022)	¥	
**Mean/Median age**	0.2827	−0.0014 (−0.0041; 0.0012)		
**Diagnostic procedure (Auscultation > Echography)**	<0.0001		*	
- Echography > Echography		0.0662 (0.0403; 0.0921)		
- Echography > No confirmation		0.0461 (−0.0080; 0.1003)		
**Diagnostic criteria (WHF)**	<0.0001			
- WHO		−0.0556 (−0.0816; −0.0297)	0.0008	−0.0402 (−0.0637; −0.0167)
- Others		−0.0896 (−0.1178; −0.0613)	< 0.0001	−0.0638 (−0.0899; −0.0377)
**WHO regions (Africa)**	<0.0001			
- Eastern Mediterranean		−0.0601 (−0.0980; −0.0222)	0.1481	−0.0437 (−0.1030; 0.0155)
- Europe		−0.0212 (−0.0810; 0.0387)	0.1539	0.0488 (−0.0183; 0.1160)
- South-East Asia		−0.0732 (−0.1023; −0.0440)	0.0165	−0.0841 (−0.1529; −0.0154)
- The Americas		0.0001 (−0.0450; 0.0453)	0.0546	0.0519 (−0.0010; 0.1049)
- Western Pacific		0.0161 (−0.0140; 0.0461)	0.8557	−0.0100 (−0.1174; 0.0975)
**UNSD of countries (Africa)**	<0.0001			
- Asia		−0.0553 (−0.0819; −0.0287)	0.1343	0.0437 (−0.0135; 0.1009)
- Europe		−0.0108 (−0.0708; 0.0491)	0.1539	0.0488 (−0.0183; 0.1160)
- Oceania		0.0270 (−0.0028; 0.0568)	0.1643	0.0784 (−0.0321; 0.1889)
- The Americas		0.0104 (−0.0345; 0.0553)	0.0546	0.0519 (−0.0010; 0.1049)
**Income (Low)**	0.0028			
- Lower-middle		−0.0561 (−0.0913; −0.0210)	0.4675	−0.0141 (−0.0522; 0.0240)
- Upper-middle		−0.0094 (−0.0492; 0.0305)	0.0150	−0.0601 (−0.1085; −0.0117)
- High		0.0020 (−0.0482; 0.0523)	0.0397	−0.0608 (−0.1188; −0.0029)
**Area (Rural)**	0.3730			
- Urban		0.0082 (−0.0355; 0.0520)		
- Both		0.0520 (−0.0165; 0.0746)		
**Setting (Community-based)**	0.8989			
- School-based		−0.0068 (−0.0425; 0.0290)		
- Both		−0.0176 (−0.1084; 0.0732)		

^¥^Not included in the multivariable model because of 29% of missing data.

^*^Successively removed from the multivariable model if p values > 0.10.

^†^Residual heterogeneity I² = 98.4%; Amount of heterogeneity accounted for R² = 57.3%.

For each meta-analysis, we found some substantial heterogeneity across the included studies overall and within subgroups (Table 1 and Supplementary Table 9 in the Appendix,). There was some evidence of publication bias across the contributing studies (Appendix, Supplementary Table 9). For overall RHD prevalence analyses, the publication bias was found for all analyses except for WHF criteria and echo > nothing procedure (Table 1, Supplementary Figs. 1–6 in the Appendix).

### Evolution of clinically silent rheumatic heart disease

Supplementary Fig. 8 in the Appendix summarizes studies reporting on the evolution of definite RHD and borderline RHD. The majority of studies were from the Western Pacific and Africa, and published from 2011 to 2017. The ages of a total of 824 participants ranged from ranged from 5–18 years. The proportion of participants on penicillin prophylaxis ranged from 18.8–100% with only three studies reporting on the adherence rate to secondary prophylaxis. The median duration of follow-up ranged from 3.7–90 months.

Table 3 depicts the evolution of definite and borderline RHD, and nonspecific valvular abnormalities. Definite RHD progressed in 7.5% (95% CI 1.5–17.6) of the cases, while 60.7% (95% CI 42.4–77.5) of cases remained stable over the course of follow-up. On the other hand, the progression rate for borderline RHD was 11.3% (95% CI 6.9–16.5). Moderate and substantial heterogeneity was noted across included studies overall, with no evidence of publication bias. Stable or progressed lesions were mostly determined by increasing age, presence of a functional aortic valve abnormality, higher durations from diagnosis, receipt of secondary prophylaxis, and presence of a pathological mitral regurgitation murmur (Fig. 3).

**Table 3:** Evolution of clinically silent rheumatic heart disease (RHD).

RHD definition	N Studies	N Participants	Prevalence, per 1000 (95%CI)	Prediction interval	I² (95%CI)	H (95%CI)	p heterogeneity	p Egger test
**Definite RHD**								
• Progression*	6	315	7.5 (1.5–17.6)	0.0–50.4	2.5 (1.7–3.6)	83.5 (65.4–92.1)	< 0.0001	0.549
• Regression**	6	315	24.6 (14.9–35.9)	1.9–61.2	1.9 (1.3–2.9)	72.8 (37.5–88.2)	0.0025	0.489
• Stable (persistence)***	6	315	60.7 (42.4–77.5)	7.0–99.7	2.9 (2.1–4.1)	88.4 (77.4–94.1)	< 0.0001	0.643
**Borderline RHD**								
• Progression^§^	7	377	11.3 (6.9–16.5)	2.0–26.8	1.4 (1.0–2.1)	46.6 (0.0–77.5)	0.0811	0.608
• Regression^§§^	6	322	39.1 (28.8–49.9)	11.0–72.0	1.7 (1.1–2.7)	67.4 (22.7–86.3)	0.0089	0.233
• Stable (persistence)^§§§^	6	322	48.6 (36.7–60.6)	14.1–84.0	1.9 (1.3–2.9)	73.5 (39.4–88.4)	0.0020	0.562
**Nonspecific valvular abnormalities (NSVA)**								
• Progression^&^	3	126	8.7 (4.4–14.2)	0.0–57.9	1.0 (1.0–1.2)	0.0 (0.0–26.4)	0.868	0.729
• Stable (persistence)^&&^	2	64	92.2 (84.5–97.5)	NA	1.0	0.0	0.710	NA

*Progression of definite RHD = Number of cases of definite RHD which progressed to clinically manifest RHD/Number of definite RHD cases at the onset of follow up; **Regression of definite RHD = Number of cases of definite RHD which converted to borderline RHD/Number of definite RHD cases at the onset of follow up; ***Stable definite RHD = Number of cases of definite RHD which remained stable during follow up/Number of definite RHD cases at the onset of follow up.

^§^Progression of borderline RHD = Number of cases of borderline RHD which progressed to definite RHD/Number of borderline RHD cases at the onset of follow up; ^§§^Regression of borderline RHD = Number of cases of borderline RHD which converted to normal/Number of borderline RHD cases at the onset of follow up; ^§§§^Stable borderline RHD = Number of cases of borderline RHD which remained stable during follow-up/Number of definite RHD cases at the onset of follow up.

^&^Progression NSVA = Number of normal cases which developed borderline or definite RHD/Number of normal cases at the onset of follow up; ^&&^Stable NSVA = Number of cases diagnosed as normal which remained normal after follow up (Stable normal)/Number of normal cases at the onset of follow up.

NA = not applicable; CI = confidence interval.

## Discussion

This systematic review and meta-analysis provides a critical summary of the global prevalence of RHD based on data pooled from 82 observational community- and school-based studies involving 1,090,792 individuals. There were several key findings: (1) we found a high overall prevalence of RHD of varying from 5.2‰ to 26.1‰ depending on diagnostic criteria and procedure used. This prevalence was highest in studies which employed WHF criteria followed by those which used the WHO criteria (21.6‰ versus 11.3‰) and was also higher with echo > echo (21.2‰) procedure compared with auscultation > echo (15.6‰) procedure. (2) The prevalence RHD varied significantly with the level of income at country level, diagnostic criteria used and by region (i.e., higher in Africa than South East Asia); and (3) The lesions of over three-quarters of persons diagnosed with definite RHD either remained stable or progressed, while 11% of those diagnosed with borderline RHD progressed to definite RHD over 3.7–90 months of follow-up.

Globally, the prevalence of RHD was about three times greater for studies which used the echo > echo procedure compared with those which used the auscultation > echo diagnostic procedure. Echocardiography has been reported to be far more sensitive than cardiac auscultation in screening for RHD^43,50,56^. Marijon reported a failure of auscultation to detect more than 90% of RHD cases detected with echocardiography^38^. Auscultation is therefore an ineffective screening method for RHD, especially in endemic regions. This finding in accordance with endorsement of echocardiography as a screening tool for RHD in endemic areas by the WHO^104^. In this light, a number of studies have evaluated the sensitivity of a more conducive echocardiographic screening tool, the handheld echocardiography (HAND), which has demonstrated its superiority over auscultation^42,76^. HAND is particular useful as it is associated with an acceptable sensitive and specificity for both borderline and definite RHD, and a sensitivity of greater than 90% of definite RHD when used by nurses^77^, and an excellent sensitivity and specificity when used by experienced physicians^81,98^. In addition, it is less costly, and portable when compared with the standard echocardiography. This is critical in overcoming the limitation of large-scale echocardiographic screening in resource-limited settings.

The prevalence of RHD was over two times greater for studies employing the WHF diagnostic criteria than those using the WHO criteria. This discrepancy could be explained by: a difference in definition for case detection and diagnostic criteria for RHD^99,104^; the difficulties associated with large-scale screening; method of screening; and the period during which data was collected for the included studies. These findings could signify that the true prevalence of RHD is underrated or overrated by studies employing the WHO or WHF criteria, respectively. Indeed, the prevalence of definite RD was about twice greater for WHF studies than WHO studies. Furthermore, the prevalence of borderline RHD was over three times greater than that of probable RHD. The major reasons for moderate to high risk of bias in the prevalence of RHD from studies included in this systematic review and meta-analysis were: the use of auscultation in screening for RHD, non-randomized sampling, and failure to report on the study setting, appropriate numerator (number of cases of RHD) to compute the prevalence of RHD; the use of an acceptable case definition for RHD or study setting.

The relatively high prevalence of borderline RHD could be due to a high false-positive rate associated with the WHF criteria^76^. It is noteworthy that the clinical significance of borderline RHD in individuals with no prior history of ARF remains obscure. In fact, the WHF affirms that the recent criteria was “established to improve sensitivity at the expense of specificity,” and does not necessarily represent a diseased state^7^. Roberts *et al*.^76^ in 2014 after screening a group of 3946 and 1053 children considered to be at high- and low-risk of RHD respectively, demonstrated that borderline RHD could be found in about 0.5% of the low-risk population. High positive rates are crucial because it may lead to unnecessary health expenditures on the part of individual and his/her family as they might be dealing with the wrong diagnosis of a chronic disease. Also, this further weighs on the healthcare system as there is the need to allocate adequate resources required for further evaluation of such potential cases, which is usually difficult in resource-poor settings. The high false-positive rate of the WHF diagnostic criteria cannot be completely disregarded due to its stronger association with populations at high-risk, compared with those at low-risk of RHD^76^.

There was no significant difference in the prevalence of RHD among school-based compared with community-based studies, and did not influence the variability of the prevalence of RHD globally. This is contrary to the hypothesis that school-based studies are likely to underrate the actual burden of RHD due to the association between school attendance and socioeconomic status; which in turn, is a principal risk factor for RHD^100^. Our finding ties with that of Rothenbühler *et al*. where no significant difference in the prevalence of RHD was noted among children sampled in schools and the community^9^. However, with the inclusion of an unbalanced proportion of studies which used community- (64/82, 78%) compared with school-based interventions, this claim warrants further investigation. Engel *et al*.^30^, 2015 and Gemechu *et al*.^31^, 2017 estimated the prevalence of RHD in Ethiopian scholars and at population level respectively, two years apart. They noted a higher prevalence of RHD at population level (56.7 cases per 1000) than schools (31 cases per 1000).

The prevalence of clinically silent RHD was over twice greater than that of clinically manifest RHD. A higher ratio was noted by Rothenbühler *et al*.^9^. Asymptomatic persons with no history of acute rheumatic fever but presenting with echocardiographic signs of RHD are said to have a clinically silent RHD, which is a latent stage of RHD^101^. The sensitivity of cardiac auscultation in detecting cases of latent RHD is very low, with a greater majority of the cases detected by active surveillance using echocardiography^42,43,50,102^. The natural progression of latent RHD, which is still obscure, is associated with clinical and economic implications as to whether the affected individual should be placed on penicillin prophylaxis or not^105^. Although, it has been suggested that the progression of latent RHD can be halted by early institution of penicillin prophylaxis, this claim is yet to be confirmed by appropriate prospective studies^106,107^. The natural progression of latent RHD has been evaluated by some prospective studies^40,102,105,108^. However, appropriate comparison across cohorts is limited by small sample sizes, the use of non-standardized criteria for diagnosis of RHD, short duration of follow up, varying proportion of participants on penicillin prophylaxis and rates of adherence to penicillin prophylaxis. We noted that about 70% of children diagnosed with definite RHD either progressed or remained stable over time. It is crucial to report disease progression according to disease severity (mild, moderate and severe). Indeed, participants with moderate and/or severe definite RHD have been reported to have a greater progression rate and poorer outcome than those with mild definite RHD^109,110^. Secondary prophylaxis coupled with adherence enhancement as proposed by the WHF in 2012 is advised by some experts that individuals with moderate to severe forms of definite RHD^5^. Whether penicillin prophylaxis is able halt the progression of RHD still remains obscured and warrants further investigation^111^. In addition, about 11% of participants initially diagnosed with borderline RHD progressed to definite RHD. This finding suggests, to a minimum, that individuals with borderline disease need surveillance for disease progression as the process persists with time^112,113^.

Despite the drastic drop in the prevalence of RHD in most high-income and some low-income countries such as Cuba, it still remains high in many developing countries^5,114^. With the absence of an effective vaccine to prevent RHD, WHO experts endorsed a multilevel approach for the control and/or eradication of RHD which consists of: the improving the social economic and environmental conditions of at-risk populations, referred to as ‘primordial prevention’; treating all patients with strep throat using penicillin – ‘primary prevention’; using antibiotic prophylaxis in persons with history of rheumatic fever or RHD prevent recurrence of an hence reduce progression of already established cardiac lesions, referred to as ‘secondary prevention’; and treatment medically and/or surgically the complications of RHD, known as ‘tertiary prevention’^7,104^. However, the implementation of these recommendations faces several challenges^7^.

Though the meta-analytic techniques used in this study were robust, the findings herein should be interpreted with care taking into account the study limitations. Firstly, we noted substantial heterogeneity in the prevalence of RHD across countries and regions, however more than half of the heterogeneity was explained by the final multivariable model. Indeed, studies have shown significant differences in the prevalence of RHD, when the WHO diagnostic criteria of 2006 is compared with the WHF criteria of 2012^76^. Also, the WHF diagnostic criteria of 2012 have not been universally accepted^52,53,59^. Consequently, alternative guidelines have been adopted in many countries^115^. This might explain the heterogeneity of RHD observed across studies. Secondly, despite the superiority of echocardiography over auscultation in diagnosing RHD, there is still no gold standard for diagnosing RHD as echocardiography relies on criteria-specific sensitivity and specificity which needs to be improved. In fact, there are concerns that available echocardiographic criteria for diagnosing RHD might overestimate the actual burden of RHD^76^. Though, the age of participants included in this study spanned from 3 to 74 years, specific criteria for echo-based diagnosis of RHD and studies on the prevalence of RHD in adults, in whom we expect to be the vast majority in developing countries, are sparse. The current criteria for the diagnosis of subclinical RHD might not be appropriate in adults, as such criteria were developed and validated in children in which early diagnosis and intervention can positively impact the natural history of RHD. Thirdly, all WHO regions were not uniformly represented partly due to difficulties in retrieving the full-text of articles published in local journals. For example, there are regions represented by just a single study or two. The authors will advise the prevalence rates obtained for such regions not be taken into consideration when comparing the global prevalence of RHD according to the different WHO regions. More studies are needed in these regions reliable statistics concerning the prevalence of RHD in these regions. Finally, evidence from studies reporting on the evolution of clinically silent RHD are limited by small sample sizes, short duration of follow-up and non-standardized criteria for diagnosis and classification of RHD.

This study reveals a high prevalence of RHD among WHO regions, with the highest rates recorded in Africa. The prevalence of RHD is highest with studies employing the WHF diagnostic criteria and those which use echocardiography for both screening and confirmation of RHD. Well-designed prospective studies with longer periods of follow-up, larger sample sizes to provide adequate data on the evolution of RHD and standardization of criteria to diagnose and classify RHD, preferably in the context of randomized trials of the effectiveness of antibiotic prophylaxis, are warranted to inform the management of asymptomatic RHD detected on screening echocardiography.

## Supplementary information

Appendix

## Data Availability

All data generated or analyzed during this study are included in this published article and its Supplementary Information files.
