# Supplementary material for: Prevalence and progression of rheumatic heart disease: a global systematic review and meta-analysis of population-based echocardiographic studies
Source: Sci Rep. 2019 Nov 19;9:17022. doi: 10.1038/s41598-019-53540-4 (PMC6863880; doi:10.1038/s41598-019-53540-4)

SUPPLEMENTARY MATERIAL

Prevalence and progression of rheumatic heart disease: a global systematic review and meta-analysis of population-based echocardiographic studies

Jean Jacques Noubiap, Valirie N. Agbor, Jean Joel Bigna, Arnaud D. Kaze, Ulrich Flore Nyaga, Bongani M. Mayosi

Contents

Supplementary Table 1. Search strategy on PubMed 2

Supplementary Table 2. Search strategy on EMBASE 2

Supplementary Table 3. Search strategy on Web of Science 2

Supplementary Table 4. Search strategy on Global Index Medicus 3

Supplementary Table 5. Search strategy on Cochrane Library 3

Supplementary Table 6. Characteristics of included prevalence studies 4

Supplementary Table 7. Individual characteristics of included studies reporting on the prevalence of rheumatic heart disease 5

Supplementary Table 8. Individual characteristics of included studies reporting on the evolution of clinically rheumatic heart disease 9

Supplementary Table 9. Summary statistics from meta-analyses of prevalence studies on rheumatic heart disease, by subgroup 10

Supplementary Figure 1. Funnel plot for publication bias for studies using the WHO diagnostic criteria 13

Supplementary Figure 2. Funnel plot for publication bias for studies using the WHF diagnostic criteria 14

Supplementary Figure 3. Funnel plot for publication bias for studies using diagnostic criteria other than WHF and WHO 15

Supplementary Figure 4. Funnel plot for publication bias for studies using an auscultation-echocardiographic screening protocol 16

Supplementary Figure 5. Funnel plot for publication bias for studies using an echocardiography-echocardiography screening protocol 17

Supplementary Figure 6. Funnel plot for publication bias for studies using only one step echocardiography screening protocol 18

Supplementary Table 1. Search strategy on PubMed

	Search terms	Items found
#1	("Rheumatic Fever"[Mesh] OR "Rheumatic Heart Disease"[Mesh] OR "Disease, Rheumatic Heart"[Mesh] OR "Diseases, Rheumatic Heart"[Mesh] OR "Heart Disease, Rheumatic"[Mesh] OR "Heart Diseases, Rheumatic"[Mesh] OR "Rheumatic Heart Diseases"[Mesh] OR "Bouillaud Disease"[Mesh] OR "Disease, Bouillaud"[Mesh] OR "Bouillaud's Disease"[Mesh] OR "Bouillauds Disease"[Mesh] OR "Disease, Bouillaud's"[Mesh] OR "Fever, Rheumatic"[Mesh] OR "Fevers, Rheumatic"[Mesh] OR "Rheumatic Fevers"[Mesh] OR "Rheumatic Fever, Acute"[Mesh] OR Rheumatic Fever[tiab] OR Rheumatic Heart Disease[tiab] OR Rheumatic Heart[tiab] OR Rheumatic Heart[tiab] OR Rheumatic[tiab] OR Bouillaud[tiab] OR rheumatic valvular heart disease[tiab] OR "RHD" OR RHD[tiab])	57,953
#2	("Echocardiography"[Mesh] OR Echocardiography[tiab] OR echocardio* OR "cardiac ultrasound" OR "cardiac echo*" OR "cardiac echography")	174,373
#3	#1 AND #2	2,884

Supplementary Table 2. Search strategy on EMBASE

#1	('rheumatic heart disease'/exp OR 'rheumatic fever'/exp OR 'rheumatic heart disease':ab,ti OR 'rheumatic cardiac':ab,ti OR 'rheumatic heart':ab,ti OR 'rheumatic fever':ab,ti OR 'rheumatic valve':ab,ti OR 'rheumatic valvular':ab,ti OR Bouillaud:ab,ti OR Bouillauds:ab,ti OR 'rheumatic cardiac disease':ab,ti OR rhd:ab,ti OR 'rheumatic valvular heart disease':ab,ti)	31,390
#2	('echocardiography'/exp OR 'cardiac echography':ab,ti OR echocardiogram:ab,ti OR 'heart echography':ab,ti)	278,025
#3	#1 AND #2	3,324
#4	#3 AND [embase]/lim NOT [medline]/lim	1,169

Supplementary Table 3. Search strategy on Web of Science

	Search terms	Items found
#1	(((((((('rheumatic heart disease' OR 'rheumatic fever') OR 'rheumatic cardiac') OR 'rheumatic valve') OR 'rheumatic valvular) OR Bouillaud) OR bouillauds) OR 'rheumatic cardiac disease') OR rhd) OR 'rheumatic valvular heart disease')	37,399
#2	('echocardiography' OR 'cardiac echography' OR echocardiogram OR 'heart echography')	235,302
#3	#1 AND #2	3,471
#4	Medline excluded	2,567

Web of Science includes Web of Sciences Collection, Current Contents Connect, SciELO citation Index, Russian Science Citation Index, and KCI-Korean Journal Database

Supplementary Table 4. Search strategy on Global Index Medicus

#1	tw:('echocardiography' OR 'cardiac echography' OR echocardiogram OR 'heart echography') AND (instance:"ghl")	**1,029,812**
**#2**	tw:('rheumatic heart disease' OR 'rheumatic fever' OR 'rheumatic cardiac' OR 'rheumatic valve' OR 'rheumatic valvular' OR 'bouillaud' OR 'bouillauds' OR 'rheumatic cardiac disease' OR 'rhd' OR 'rheumatic valvular heart disease') AND (instance:"ghl")	**2,700**
**#3**	**#1 AND #2**	**370**
**#4**	**Medline excluded**	**1**

Global Index Medicus: WPRIM (Western Pacific), LILACS (Americas), IMERMR (Eastern Mediterranean), AIM (Africa), WHOLIS (KMS), BBO – Dentistry (Brazil), IMSEAR (South-East Asia)

Supplementary Table 5. Search strategy on Cochrane Library

#1	'echocardiography' OR 'cardiac echography' OR echocardiogram OR 'heart echography'	**9,136**
**#2**	'rheumatic heart disease' OR 'rheumatic fever' OR 'rheumatic cardiac' OR 'rheumatic valve' OR 'rheumatic valvular' OR 'bouillaud' OR 'bouillauds' OR 'rheumatic cardiac disease' OR 'rhd' OR 'rheumatic valvular heart disease'	**583**
**#3**	**#1 AND #2**	**75**

Supplementary Table 6. Characteristics of included prevalence studies

Characteristics	N = 82 studies
Setting	
- Community-based	14
- School-based	64
- Both	2
- Not reported	2
Area	
- Urban	33
- Rural	16
- Both	26
- Not reported	9
Sampling method	
- Random	80
- Not reported	2
Diagnostic criteria	
- WHO	30
- WHF	32
- Others	22
Diagnostic procedure	
- Auscultation>Echo	31
- Echo>Echo	46
- Echo>Nothing	5
Endemic area	
- Yes	69
- No	13
WHO regions	
- Africa	22
- The Americas	6
- Eastern Mediterranean	9
- Europe	3
- South-East Asia	22
- Western Pacific	20
UNSD regions	
- Africa	25
- The Americas	6
- Asia	29
- Europe	3
- Oceania	19
Income	
- Low	20
- Lower-Middle	34
- Upper-Middle	19
- High	9

Auscultation>Echo = Auscultation only for screening followed with echography confirmation; Echo>Echo: Echography +/- auscultation for screening followed with echography for confirmation; Echo>Nothing: Echography only for screening without echography confirmation; GDP = Gross national income per capita; HDI = Human Development Index; WHO: World Health Organization; WHF: World Heart Federation; UNSD: United Nations Statistic Division

Supplementary Table 7. Individual characteristics of included studies reporting on the prevalence of rheumatic heart disease

Study	Bias	Criteria	Year	Country	Period	Endemic area	GDP	HDI	GINI (*100)	WHO	UNSD	Level of income	Design	Population	Setting	Random sampling	Area	Diagnostic criteria	Male, %	Age, years	Age range	Sample
**Abdel-Moula 1998**	Low	Others	1998	Egypt	NR	Yes	1042	0.691	31.8	Eastern Mediterranean	Africa	2.Lower-Middle	Cross-sectional	School children	School-based	Yes	Urban	Auscultation>Echo				5465
**Ahmed 2005**	Moderate	Others	2005	Bangladesh	1991	Yes	285	0.579	32.1	South-East Asia	Asia	2.Lower-Middle	Cross-sectional	Children	Both	Yes	Rural	Echo>Echo		12.8	5;15	5923
**Allen 2017**	Low	WHF	2017	Samoa	2013; 2015	Yes	4179	0.704	42	Western Pacific	Oceania	3.Upper-Middle	Cross-sectional	School children	School-based	Yes	NR	Echo>Nothing	48.90	10.2	5;17	11434
**Al-Munibari 2001**	Moderate	Others	2001	Yemen	1997-1998	Yes	396	0.482	36.7	Eastern Mediterranean	Asia	2.Lower-Middle	Cross-sectional	School children	School-based	Yes	Both	Auscultation>Echo	49.90		5;18	5000
**Anabwani 1996**	Moderate	Others	1996	Kenya	NR	Yes	260	0.555	48.5	Africa	Africa	2.Lower-Middle	Cross-sectional	School children	NR	NR	Rural	Echo>Nothing				1115
**Baroux 2013**	Low	Others	2013	New Caledonia	2008; 2010	Yes	12580	NR	NR	Western Pacific	Oceania	4.High	Cross-sectional	School children	School-based	Yes	Urban	Echo>Echo	50.20	9.6	5;15	12803
**Ba-Saddik 2012**	Low	WHO	2012	Yemen	2004; 2005	Yes	754	0.482	36.7	Eastern Mediterranean	Asia	2.Lower-Middle	Cross-sectional	School children	School-based	Yes	Both	Auscultation>Echo	51.00	11.2	5;16	6000
**Beaton 2012**	Low	WHO	2012	Uganda	2010	Yes	595	0.493	41	Africa	Africa	1.Low	Cross-sectional	School children	School-based	Yes	Urban	Echo>Echo	44.00	9.6	5;16	4869
**Beaton 2015**	Low	WHF	2015	Uganda	NR	Yes	655	0.493	41	Africa	Africa	1.Low	Cross-sectional	School children	School-based	Yes	NR	Echo>Echo	47.00	10.8		1420
**Bhardwaj 2012**	Moderate	Others	2012	India	NR	Yes	1346	0.624	35.2	South-East Asia	Asia	2.Lower-Middle	Cross-sectional	General population	Community-based	Yes	Rural	Auscultation>Echo	48.00	30.4		1882
**Bhaya 2010**	Low	WHO	2010	India	2007; 2008	Yes	1036	0.624	35.2	South-East Asia	Asia	2.Lower-Middle	Cross-sectional	School children	School-based	Yes	Urban	Echo>Echo			6;15	1059
**Bodian 2014**	Low	WHO	2014	Senegal	2011	Yes	1082	0.494	40.3	Africa	Africa	1.Low	Cross-sectional	School children	School-based	Yes	Urban	Echo>Echo	60.10	9.7	5;18	2019
**Campanale 2017**	Low	WHF	2017	Madagascar	2013; 2015	Yes	440	0.512	42.7	Africa	Africa	1.Low	Cross-sectional	General population	Community-based	Yes	Both	Echo>Echo			≥ 20	337
**Campanale 2017**	Low	WHF	2017	Madagascar	2013; 2015	Yes	440	0.512	42.7	Africa	Africa	1.Low	Cross-sectional	General population	Community-based	Yes	Both	Echo>Echo			5;19	522
**Carapetis 2008**	Low	WHO	2008	Tonga	2003;2004	Yes	2157	0.721	37.5	Western Pacific	Oceania	3.Upper-Middle	Cross-sectional	School children	School-based	Yes	Urban	Auscultation>Echo	52.00	8.0	3;15	4794
**Chaikitpinyo 2014**	Moderate	Others	2014	Thailand	2006	No	3369	0.74	37.8	South-East Asia	Asia	3.Upper-Middle	Cross-sectional	School children	School-based	Yes	Both	Auscultation>Echo	50.90	10.0	5;15	8555
**Colquhoun 2014**	Moderate	WHO	2014	Fiji	2009; 2010	Yes	3511	0.736	36.4	Western Pacific	Oceania	3.Upper-Middle	Cross-sectional	School children	School-based	Yes	Urban	Echo>Echo			5;14	1666
**Colquhoun 2014**	Moderate	WHF	2014	Fiji	2009; 2011	Yes	3652	0.736	36.4	Western Pacific	Oceania	3.Upper-Middle	Cross-sectional	School children	School-based	Yes	Urban	Echo>Echo			5;15	1666
**Cramp 2012**	Low	Others	2012	New Zealand	2009	No	28201	0.915	NR	Western Pacific	Oceania	4.High	Cross-sectional	School children	School-based	Yes	Both	Echo>Echo			5;17	685
**El-Aroussy 2013**	Low	WHO	2013	Egypt	2009; 2011	Yes	2602	0.691	31.8	Eastern Mediterranean	Africa	2.Lower-Middle	Cross-sectional	School children	School-based	Yes	Urban	Auscultation>Echo	49.10	11.1	6;18	48930
**Engel 2015**	Low	WHF	2015	Ethiopia	2008; 2012	Yes	380	0.448	33.2	Africa	Africa	1.Low	Cross-sectional	School children	School-based	Yes	Rural	Echo>Echo	49.00	10.7	5;20	2000
**Engel 2015**	Low	WHF	2015	South Africa	2008; 2012	Yes	7362	0.666	63.4	Africa	Africa	3.Upper-Middle	Cross-sectional	School children	School-based	Yes	Rural	Echo>Echo	41.00	12.2	4;24	2720
**Engelman 2016**	Moderate	WHF	2016	Fiji	2012	Yes	4547	0.736	36.4	Western Pacific	Oceania	3.Upper-Middle	Cross-sectional	School children	School-based	Yes	Urban	Echo>Echo	48.60	10.0	5;15	2004
**Fakakovikaetau 2016**	Low	WHF	2016	Tonga	2008; 2015	Yes	4273	0.721	37.5	Western Pacific	Oceania	3.Upper-Middle	Cross-sectional	Children	Community-based	Yes	Both	Echo>Echo			5;14	36990
**Farrag 2014**	Low	WHO	2014	Egypt	NR	Yes	3181	0.691	31.8	Eastern Mediterranean	Africa	2.Lower-Middle	Cross-sectional	School children	School-based	Yes	NR	Echo>Echo	49.10		6;18	48930

Study	Bias	Criteria	Year	Country	Period	Endemic area	GDP	HDI	GINI (*100)	WHO	UNSD	Level of income	Design	Population	Setting	Random sampling	Area	Diagnostic criteria	Male, %	Age, years	Age range	Sample
**Gautam 2014**	High	WHO	2014	Nepal	2006; 2007	Yes	8226	0.558	32.8	South-East Asia	Asia	1.Low	Cross-sectional	School children	School-based	Yes	NR	Auscultation>Echo			5;15	2043
**Gemechu 2017**	Low	WHF	2017	Ethiopia	NR	Yes	660	0.448	33.2	Africa	Africa	1.Low	Cross-sectional	General population	Community-based	Yes	Rural	Echo>Nothing	46.00	13.2	6;25	987
**Gul 2009**	Low	WHO	2009	Pakistan	2006	Yes	874	0.55	30.7	Eastern Mediterranean	Asia	2.Lower-Middle	Cross-sectional	School children	School-based	Yes	Urban	Auscultation>Echo		13.3	6;16	1773
**Huang 2017**	Low	WHF	2017	Samoa	NR	Yes	4149	0.704	42	Western Pacific	Oceania	3.Upper-Middle	Cross-sectional	School children	School-based	Yes	Both	Echo>Echo	48.00	11.3	5;18	1058
**Jose 2003**	Moderate	Others	2003	India	2001; 2002	Yes	457	0.624	35.2	South-East Asia	Asia	2.Lower-Middle	Cross-sectional	School children	School-based	Yes	Rural	Auscultation>Echo	51.07		6;18	229829
**Kane 2013**	Moderate	Others	2013	Senegal	2010	Yes	1002	0.494	42	Africa	Africa	1.Low	Cross-sectional	School children	School-based	Yes	Urban	Echo>Echo	51.60		5;18	2004
**Kaul 2005**	Low	WHO	2005	India	1999; 2000	Yes	439	0.624	35.2	South-East Asia	Asia	2.Lower-Middle	Cross-sectional	School children	School-based	Yes	Rural	Auscultation>Echo			5;15	4125
**Kennedy 2014**	Low	WHF	2014	Solomon Islands	NR	Yes	1859	0.515	37	Western Pacific	Oceania	2.Lower-Middle	Cross-sectional	School children	School-based	Yes	Urban	Echo>Echo			5;15	700
**Kumari 2013**	Low	WHO	2013	India	2011	Yes	1462	0.624	35.2	South-East Asia	Asia	2.Lower-Middle	Cross-sectional	School children	School-based	Yes	Both	Echo>Echo	53.05		5;16	4213
**Kwok 2015**	Low	WHF	2015	Angola	2013	Yes	2760	0.533	42.7	Africa	Africa	2.Lower-Middle	Cross-sectional	Children	Community-based	Yes	Both	Echo>Echo	44.20	10.1	4;20	574
**Ledos 2015**	Low	WHF	2015	New Caledonia	2010	Yes	12580	NR	NR	Western Pacific	Oceania	4.High	Cross-sectional	Young adults	Community-based	Yes	Urban	Echo>Nothing	55.00	20.0	18;22	834
**Longo-Mbenza 1998**	Low	WHO	1998	Congo	1996	Yes	450	0.592	48.9	Africa	Africa	1.Low	Cross-sectional	School children	School-based	Yes	Both	Echo>Echo		10.6	5;16	4848
**Lu 2015**	Moderate	WHF	2015	Uganda	NR	Yes	655	0.493	41	Africa	Africa	1.Low	Cross-sectional	School children	School-based	Yes	Both	Echo>Echo	47.00	10.8	5;17	4773
**Mahmoudi 2003**	Low	WHO	2003	Iran	NR	No	1891	0.774	38.8	Eastern Mediterranean	Asia	3.Upper-Middle	Cross-sectional	School children	School-based	Yes	Urban	Auscultation>Echo	62.10		7;18	167786
**Marijon 2007**	Low	WHO	2007	Cambodia	2001; 2002	Yes	330	0.563	30.76	Western Pacific	Asia	2.Lower-Middle	Cross-sectional	School children	School-based	Yes	Urban	Auscultation>Echo	52.00	11.7	6;17	3677
**Marijon 2007**	Moderate	WHO	2007	Mozambique	2005	Yes	350	0.418	45.6	Africa	Africa	1.Low	Cross-sectional	School children	School-based	Yes	Urban	Auscultation>Echo	47.50	10.6	6;17	2170
**Mirabel 2015**	Low	WHF	2015	New Caledonia	2008; 2011	Yes	12580	NR	NR	Western Pacific	Oceania	4.High	Cross-sectional	School children	School-based	Yes	Urban	Echo>Echo			9;10	17633
**Misra 2007**	Low	Others	2007	India	NR	Yes	707	0.624	35.2	South-East Asia	Asia	2.Lower-Middle	Cross-sectional	School children	NR	NR	NR	Auscultation>Echo	57.80		4;18	118212
**Mucumbitsi 2017**	Moderate	WHF	2017	Rwanda	NR	Yes	710	0.498	50.4	Africa	Africa	1.Low	Cross-sectional	School children	School-based	Yes	Urban	Echo>Echo		11.2	6;16	2501
**Mulatu 2015**	Low	WHO	2015	Ethiopia	2013	Yes	470	0.448	33.2	Africa	Africa	1.Low	Cross-sectional	School children	School-based	Yes	Rural	Auscultation>Echo	48.99	8.9	5;15	1874
**Nair 2015**	Moderate	WHF	2015	India	2013; 2014	Yes	1513	0.624	35.2	South-East Asia	Asia	2.Lower-Middle	Cross-sectional	School children	School-based	Yes	Urban	Auscultation>Echo	64.80	12.6	5;15	2060
**Nascimento 2016**	Low	WHF	2016	Brazil	2014; 2015	No	10392	0.754	51.3	The Americas	The Americas	3.Upper-Middle	Cross-sectional	School children	School-based	Yes	Urban	Auscultation>Echo	41.00	11.9		5996
**Negi 2013**	Low	WHO	2013	India	2007; 2008	Yes	1005	0.624	35.2	South-East Asia	Asia	2.Lower-Middle	Cross-sectional	School children	School-based	Yes	Both	Auscultation>Echo	59.00	11.1	5;15	15145
**Ngaïdé 2015**	Moderate	WHF	2015	Senegal	2011	Yes	1082	0.494	40.3	Africa	Africa	1.Low	Cross-sectional	School children	School-based	Yes	Urban	Echo>Echo	60.10	9.7	5;18	2019
**Oli 1999**	Low	Others	1999	Ethiopia	NR	Yes	140	0.448	33.2	Africa	Africa	1.Low	Cross-sectional	School children	School-based	Yes	Urban	Auscultation>Echo				9388
**Paar 2010**	Low	WHO	2010	Nicaragua	2006; 2009	Yes	8724	0.645	46.6	The Americas	The Americas	2.Lower-Middle	Cross-sectional	Adults	Community-based	Yes	Both	Echo>Echo	38.00	27.4	20;35.9	489
**Paar 2010**	Moderate	WHO	2010	Nicaragua	2006; 2009	Yes	8724	0.645	46.6	The Americas	The Americas	2.Lower-Middle	Cross-sectional	School children	School-based	Yes	Both	Echo>Echo	51.00	9.5	5;15	3150

Study	Bias	Criteria	Year	Country	Period	Endemic area	GDP	HDI	GINI (*100)	WHO	UNSD	Level of income	Design	Population	Setting	Random sampling	Area	Diagnostic criteria	Male, %	Age, years	Age range	Sample
**Periwal 2006**	Low	Others	2006	India	NR	Yes	621	0.624	35.2	South-East Asia	Asia	2.Lower-Middle	Cross-sectional	School children	School-based	Yes	Urban	Auscultation>Echo	61.00		5;14	3002
**Ploutz 2016**	Moderate	WHF	2016	Uganda	2014	Yes	703	0.493	41	Africa	Africa	1.Low	Cross-sectional	School children	School-based	Yes	NR	Echo>Echo	42.10	11.1	5;17	956
**Poyyamozhi 2016**	Moderate	Others	2016	India	2005; 2006	Yes	750	0.624	35.2	South-East Asia	Asia	2.Lower-Middle	Cross-sectional	School children	Community-based	Yes	Urban	Auscultation>Echo	55.00		5;17	7137
**Regmi 1997**	Moderate	WHO	1997	India	NR	Yes	370	0.624	35.2	South-East Asia	Asia	2.Lower-Middle	Cross-sectional	School children	School-based	Yes	Urban	Auscultation>Echo			5;16	4736
**Rizvi 2003**	Moderate	Others	2003	Pakistan	1993; 1994	Yes	437	0.55	30.7	Eastern Mediterranean	Asia	2.Lower-Middle	Cross-sectional	General population	Community-based	Yes	Rural	Auscultation>Echo	53.10			9483
**Roberts 2014**	Low	WHF	2014	Australia	2008; 2010	No	40911	0.939	34.7	Western Pacific	Oceania	4.High	Cross-sectional	General population	Community-based	Yes	Rural	Echo>Echo	50.90	9.3	5;15	3946
**Roberts 2014**	Moderate	WHF	2014	Australia	2008; 2010	No	40911	0.939	34.7	Western Pacific	Oceania	4.High	Cross-sectional	School children	School-based	Yes	Urban	Echo>Echo	48.80	9.4	5;15	1053
**Rossi 2014**	Low	WHF	2014	Italy	NR	No	34814	0.887	34.7	Europe	Europe	4.High	Cross-sectional	School children	School-based	Yes	NR	Echo>Echo	54.10	16.7		684
**Sadiq 2009**	Low	Others	2009	Pakistan	2001; 2002	Yes	506	0.55	30.7	Eastern Mediterranean	Asia	2.Lower-Middle	Cross-sectional	School children	School-based	Yes	Urban	Auscultation>Echo	38.50	10.7	5;15	24980
**Sadoh 2013**	Moderate	WHO	2013	Nigeria	2011; 2012	Yes	2876	0.527	43	Africa	Africa	2.Lower-Middle	Cross-sectional	School children	School-based	Yes	Urban	Auscultation>Echo	48.98	8.9	5;15	1764
**Sanyahumbi 2016**	Moderate	WHF	2016	Malawi	2014	Yes	350	0.476	46.1	Africa	Africa	1.Low	Cross-sectional	Children	Both	Yes	Both	Echo>Echo	47.17		5;16	1450
**Satupaitea 2012**	Moderate	WHF	2012	Samoa	2008; 2012	Yes	3453	0.704	42	Western Pacific	Oceania	3.Upper-Middle	Cross-sectional	School children	School-based	Yes	Both	Echo>Echo			3;16	8457
**Saxena 2011**	Low	WHO	2014	India	2008; 2010	Yes	1090	0.624	35.2	South-East Asia	Asia	2.Lower-Middle	Cross-sectional	School children	School-based	Yes	Rural	Echo>Echo	52.65	10.8	5;15	6270
**Saxena 2017**	Low	WHF	2017	India	2010; 2013	Yes	1447	0.624	35.2	South-East Asia	Asia	2.Lower-Middle	Cross-sectional	School children	School-based	Yes	Both	Echo>Echo	55.10	10.8	5;15	16294
**Schaffer 2003**	Low	Others	2003	USA	1993; 1995	No	27777	0.92	41	The Americas	The Americas	4.High	Cross-sectional	Adults	Community-based	Yes	Rural	Echo>Echo			45;74	3501
**Shrestha 2016**	Moderate	WHF	2016	Nepal	2012; 2014	Yes	10719	0.558	32.8	South-East Asia	Asia	1.Low	Cross-sectional	School children	School-based	Yes	Both	Echo>Echo	51.70	10.0	5;15	5178
**Singh 2014**	Low	WHF	2014	India	NR	Yes	1447	0.624	35.2	South-East Asia	Asia	2.Lower-Middle	Cross-sectional	School children	School-based	Yes	Both	Echo>Echo	52.77	11.2	5;15	3015
**Spitzer 2015**	Low	WHF	2015	Peru	2014	No	6491	0.74	44.3	The Americas	The Americas	3.Upper-Middle	Cross-sectional	School children	School-based	Yes	Both	Echo>Echo	50.00	11.0	6;15	1023
**Spitzer 2015**	Low	WHO	2015	Peru	2014	No	6491	0.74	44.3	The Americas	The Americas	3.Upper-Middle	Cross-sectional	School children	School-based	Yes	Both	Echo>Echo	50.00	11.0	6;15	1023
**Sriharibabu 2013**	Moderate	Others	2013	India	2007; 2012	Yes	1236	0.624	35.2	South-East Asia	Asia	2.Lower-Middle	Cross-sectional	Children and Adults	Community-based	Yes	Rural	Auscultation>Echo	30.00	33.0	15;64	44164
**Steer 2009**	Moderate	WHO	2009	Fiji	2006	Yes	3750	0.736	36.4	Western Pacific	Oceania	3.Upper-Middle	Cross-sectional	School children	School-based	Yes	Urban	Auscultation>Echo			5;15	3462
**Thakur 1996**	Low	WHO	1996	India	1992; 1993	Yes	306	0.624	35.2	South-East Asia	Asia	2.Lower-Middle	Cross-sectional	School children	School-based	Yes	Both	Auscultation>Echo	54.00		5;16	15080
**Uner 2009**	Moderate	Others	2009	Turkey	2003; 2004	No	5380	0.767	41.2	Europe	Europe	3.Upper-Middle	Cross-sectional	School children	School-based	Yes	Urban	Auscultation>Echo	55.00	10.0	6;15	6035
**Viali 2016**	Low	WHF	2016	Samoa	2013	Yes	4219	0.704	42	Western Pacific	Oceania	3.Upper-Middle	Cross-sectional	School children	School-based	Yes	NR	Echo>Echo			14;18	5197
**Webb 2011**	Low	WHO	2011	New Zealand	NR	No	28201	0.915	NR	Western Pacific	Oceania	4.High	Cross-sectional	School children	School-based	Yes	Urban	Echo>Echo	56.00		10;13	1142
**Yadav 2010**	Moderate	WHO	2010	India	2010	Yes	1346	0.624	35.2	South-East Asia	Asia	2.Lower-Middle	Cross-sectional	School children	School-based	Yes	Urban	Auscultation>Echo	62.10		5;16	9526
**Yadeta 2016**	Low	WHF	2016	Ethiopia	2013; 2014	Yes	550	0.448	33.2	Africa	Africa	1.Low	Cross-sectional	School children	School-based	Yes	Both	Echo>Echo	52.00	13.2	6;18	3238

Study	Bias	Criteria	Year	Country	Period	Endemic area	GDP	HDI	GINI (*100)	WHO	UNSD	Level of income	Design	Population	Setting	Random sampling	Area	Diagnostic criteria	Male, %	Age, years	Age range	Sample
**Yildiz 2016**	Low	WHO	2016	Turkey	NR	No	12127	0.767	41.2	Europe	Europe	3.Upper-Middle	Cross-sectional	School children	School-based	Yes	NR	Echo>Nothing	50.66		5;15	5440
**Zaman 2015**	Moderate	Others	2015	Bangladesh	2005	Yes	484	0.579	32.1	South-East Asia	Asia	2.Lower-Middle	Cross-sectional	School children	School-based	Yes	Both	Echo>Echo	51.00	11.0	5;19	56827
**Zhimin 2006**	Low	Others	2006	China	2001; 2002	Yes	1101	0.738	42.2	Western Pacific	Oceania	3.Upper-Middle	Cross-sectional	Adults	Community-based	Yes	Both	Echo>Echo		48.3	27;71	8080

WHF: World Heart Federation; WHO: World Heart Organization; UNSD: United Nations Statistical Divisions; Auscultation>Echo = Auscultation only for screening followed with echography confirmation; Echo>Echo: Echography +/- auscultation for screening followed with echography for confirmation; E>N: Echography only for screening without echography confirmation; NR = not reported; GDP: gross domestic per capita; HDI: human development index

Supplementary Table 8. Individual characteristics of included studies reporting on the evolution of clinically rheumatic heart disease

Study, year of publication	Continent	Region	Country	WHO Region	Area	Median duration of follow up (months)	Study design	Data collection	Sampling method	Male (%)	Mean or Median age (years)	Age range (years)	Sample size	Proportion of participants on BPG	Adherence (%)	Number of cases at onset of follow-up			Predictors of stable and/progressed lesions
																Definite RHD	Borderline	NSVA	
**Beaton 2014**	Africa	East	Uganda	Africa	Both	25	Cohort	Prospective	Consecutive	40	9.6	5;13	60	23	85.3	8	43	9	Younger children; Increasing morphologic changes; Higher ASLO titres
**Beaton 2017**	Africa	East	Uganda	Africa	Both	27.6	Cohort	Prospective	Consecutive	30.6	12	10;14	227	49.3	82.4	63	164	.	Younger children (AOR = 0.83); Presence of a functional AV abnormality (AOR= 5.37)
**Bertaina 2016**	Europe	North	New Caledonia	Western Pacific	Urban	23	Cohort	Prospective	NR	32	9.8	NR	25	NR	NR	0	25	0	NR
**Bhaya 2011**	Asia	Southeast	India	Southeast Asia	NR	24	Cohort	Prospective	NR	NR	NR	6;15	39	100	NR	0	39	0	NR
**Engelman 2016**	Oceania	North	Fiji	Western Pacific	Both	90	Cohort	Prospective	Consecutive	32.6	17	8.5;12.0	98	59.2	2	20	17	21	Cases with a duration of diagnosis of at least five years
**Mirabel 2015**	Europe	West	New Caledonia	Western Pacific	Both	31	Cohort	Prospective	Consecutive	47.4	9.9	9;10	114	88.6	NR	114	0	0	NR
**Reymond 2015**	Oceania	South	Australia	Western Pacific	Both	3.7	Cohort	Prospective	Consecutive	41	13.7	8;18	117	18.8	NR	0	55	62	Borderline RHD of the MV; Borderline RHD of the AV; Non-specific valvular abnormalities; In receipt of secondary antibiotic prophylaxis
**Saxena 2011**	Asia	Southeast	India	Southeast Asia	Rural	15.4	Cohort	Prospective	NR	52.7	10.8	5;15	100	NR	NR	100	0	0	NR
**Zuhlke 2016**	Africa	South	South Africa	Africa	Urban	60.8	Cohort	Prospective	Consecutive	14	13.8	NR	44	NR	NR	10	34	0	Presence of pathological MR

ASLO = Antistreptolysin O antigen; AOR = Adjusteed odd’s ratio; AV = Aortic valve; MR = Mitral regurgitation; BGP = Benzanthine penicillin G; RHD = Rheumatic heart disease; NSVA = Non-specific valvular abnormality; NR = Not reported

Supplementary Table 9. Summary statistics from meta-analyses of prevalence studies on rheumatic heart disease, by subgroup

Subgroups	Criteria	N Studies	N Participants	Prevalence, per 1000 (95%CI)	Prediction interval	I² (95%CI)	H (95%CI)	p heterogeneity	p Egger test	p difference subgroups
Income										
**Low**	**WHF**	12	25381	31.0 (18.2-46.9)	0.0-113.3	97.6 (96.8-98.2)	6.5 (5.6-7.5)	< 0.0001	0.288	0.003
**Lower-Middle**		5	22643	10.9 (5.8-17.7	0.0-43.1	89.8 (78.9-95.0)	3.1 (2.2-4.5)	< 0.0001	0.346	
**Upper-Middle**		10	76545	31.7 (21.0-44.6)	2.4-92.1	98.6 (98.1-98.9)	8.4 (7.3-9.6)	< 0.0001	0.376	
**High**		5	24150	20.8 (8.5-38.1)	0.0-114.5	97.2 (95.4-98.3)	5.9 (4.6-7.6)	< 0.0001	0.278	
**Low**	**WHO**	6	17823	9.8 (4.2-17.7)	0.0-49.1	95.0 (91.6-97.1)	4.5 (3.4-5.8)	< 0.0001	0.367	< 0.0001
**Lower-Middle**		16	174867	9.6 (5.2-15.3)	0.0-44.3	99.0 (98.7-99.1)	9.8 (8.9-10.7)	< 0.0001	0.050	
**Upper-Middle**		6	184171	13.5 (1.8-35.7)	0.0-144.8	99.5 (99.4-99.6)	14.4 (12.7-16.2)	< 0.0001	0.020	
**High**		1	1142	51.7 (39.5-65.3)	NA	NA	NA	NA	NA	
**Low**	**A>E**	4	15475	8.2 (1.7-19.3)	0.0-101.4	96.5 (93.6-98.1)	5.4 (4.0-7.3)	< 0.0001	0.821	0.737
**Lower-Middle**		21	561970	5.5 (3.0-8.6)	0.0-27.8	99.3 (99.2-99.4)	12.3 (11.5-13.1)	< 0.0001	0.022	
**Upper-Middle**		6	196628	8.6 (0.5-25.9)	0.0-117.4	99.7 (99.6-99.7)	17.6 (15.8-19.5)	< 0.0001	0.087	
**High**		0	NA	NA	NA	NA	NA	NA	NA	
**Low**	**E>E**	15	38134	23.3 (14.5-33.7)	0.0-81.8	97.4 (96.6-98.0)	6.2 (5.4-7.0)	< 0.0001	0.317	0.212
**Lower-Middle**		12	147444	13.5 (7.6-21.0)	0.0-52.8	99.0 (98.7-99.2)	9.8 (8.8-10.9)	< 0.0001	0.008	
**Upper-Middle**		11	69884	27.2 (14.1-44.5)	0.0-118.0	99.1 (98.9-99.3)	10.8 (9.7-12.0)	< 0.0001	0.378	
**High**		8	41447	21.5 (12.7-32.5)	0.3-71.7	97.4 (96.2-98.2)	6.2 (5.2-7.4)	< 0.0001	0.066	
Endemic area										
**Endemic**	**WHF**	27	136017	26.5 (18.9-35.4)	0.4-89.1	98.8 (98.6-98.9)	9.0 (8.4-9.7)	< 0.0001	0.981	0.801
**Non endemic**		5	12702	23.9 (9.2-45.2)	0.0-141.0	97.3 (95.6-98.3)	6.1 (4.8-7.8)	< 0.0001	0.478	
**Endemic**	**WHO**	25	202612	11.2 (7.0-16.3)	0.0-48.6	98.8 (98.7-99.0)	9.3 (8.6-10.0)	< 0.0001	0.006	0.882
**Non endemic**		4	175391	12.4 (1.1-35.3)	0.0-218.1	99.2 (98.8-99.4)	10.9 (9.0-13.2)	< 0.0001	0.032	
**Endemic**	**A>E**	27	585701	6.8 (4.1-9.9)	0.0-29.2	99.3 (99.2-99.4)	11.9 (11.2-12.6)	< 0.0001	0.004	0.700
**Non endemic**		4	188372	4.5 (0.0-20.0)	0.0-175.8	99.7 (99.5-99.7)	17.1 (14.9-19.7)	< 0.0001	0.349	
**Endemic**	**E>E**	38	283852	20.9 (14.4-28.5)	0.0-88.5	99.4 (99.3-99.4)	12.7 (12.2-13.3)	< 0.0001	0.013	0.812
**Non endemic**		8	13057	22.8 (10.0-40.4)	0.0-110.1	96.8 (95.3-97.9)	5.6 (4.6-6.8)	< 0.0001	0.352	
UNSD Regions										
**Africa**	**WHF**	13	23497	32.0 (20.2-46.4)	0.5-106.5	96.8 (95.7-97.7)	5.6 (4.8-6.5)	< 0.0001	0.560	< 0.0001
**Asia**		4	26547	7.0 (4.8-9.5)	0.5-20.4	73.7 (26.4-90.6)	1.9 (1.2-3.3)	< 0.0001	0.606	
**Europe**		1	684	74.6 (56.0-95.5)	NA	NA	NA	< 0.0001	NA	
**Oceania**		12	90972	27.2 (16.5-40.5)	0.1-95.4	99.0 (98.8-99.2)	10.2 (9.2-11.3)	< 0.0001	0.647	
**The Americas**		2	7019	18.4 (0.0-73.1)	NA	98.5 (0.0-73.1)	8.3	< 0.0001	NA	

**Africa**	**WHO**	8	115404	7.1 (3.9-11.4)	0.0-26.9	97.5 (96.4-98.3)	6.3 (5.3-7.6)	< 0.0001	0.105	0.0003
**Asia**		13	241433	7.7 (2.8-14.9)	0.0-52.6	99.3 (99.2-99.4)	12.2 (11.2-13.3)	< 0.0001	0.005	
**Europe**		1	5440	5.1 (3.4-7.2)	NA	NA	NA	NA	NA	
**Oceania**		4	11064	30.8 (13.1-55-4)	0.0-207.6	97.4 (95.5-98.5)	6.2 (4.7-8.2)	< 0.0001	0.714	
**The Americas**		3	4662	29.3 (12.9-52.0)	0.0-603.4	91.3 (77.4-96.6)	3.4 (2.1-5.4)	< 0.0001	0.322	
**Africa**	**A>E**	6	69591	6.1 (2.5-11.2)	0.0-31.7	96.7 (94.8-97.9)	5.5 (4.4-7.0)	< 0.0001	0.286	< 0.0001
**Asia**		21	684195	4.9 (2.7-7.8)	0.0-23.8	99.4 (99.4-99.5)	13.3 (12.5-14.1)	< 0.0001	0.016	
**Europe**		1	6035	1.2 (0.4-2.2)	NA	NA	NA	NA	NA	
**Oceania**		2	8256	20.8 (1.6-60.1)	NA	98.9 (97.7-99.4)	9.4	< 0.0001	NA	
**The Americas**		1	5996	41.8 (36.9-47.1)	NA	NA	NA	NA	NA	
**Africa**	**E>E**	17	85180	22.5 (13.1-34.4)	0.0-95.1	98.7 (98.4-98.9)	8.8 (8.0-9.7)	< 0.0001	0.0008	< 0.0001
**Asia**		8	98779	9.2 (3.5-17.4)	0.0-52.3	99.0 (98.6-99.2)	9.8 (8.5-11.2)	< 0.0001	0.024	
**Europe**		1	684	74.6 (56.0-95.5)	NA	NA	NA	NA	NA	
**Oceania**		15	103080	27.2 (16.2-40.9)	0.0-105.9	99.2 (99.1-99.4)	11.5 (10.5-12.5)	< 0.0001	0.859	
**The Americas**		5	9186	16.4 (2.9-39.8)	0.0-160.0	97.7 (96.3-98.5)	6.6 (5.2-8.3)	< 0.0001	0.921	
WHO Regions										
**Africa**	**WHF**	13	23497	32.0 (20.2-46.4)	0.5-106.5	96.8 (95.7-97.7)	5.6 (4.8-6.5)	< 0.0001	0.560	< 0.0001
**The Americas**		2	7019	18.4 (0.0-73.1)	NA	98.5 (96.8-99.3)	8.3	< 0.0001	NA	
**Eastern Mediterranean**		0	NA	NA	NA	NA	NA	NA	NA	
**Europe**		1	684	74.6 (56.0-95.5)	NA	NA	NA	NA	NA	
**South-East Asia**		4	26547	7.0 (4.8-9.5)	0.5-20.4	73.7 (26.4-90.6)	1.9 (1.2-3.3)	0.0096	0.606	
**Western Pacific**		12	90972	27.2 (6.5-4.5)	0.1-95.4	99.0 (98.8-99.2)	10.2 (9.2-11.3)	< 0.0001	0.647	
**Africa**	**WHO**	6	17544	9.1 (3.3-17.6)	0.0-52.9	95.8 (93.1-97.5)	4.9 (3.8-6.3)	< 0.0001	0.299	< 0.0001
**The Americas**		3	4662	29.3 (12.9-52.0)	0.0-603.4	91.3 (77.4-96.6)	3.4 (2.1-5.4)	< 0.0001	0.322	
**Eastern Mediterranean**		5	273419	5.8 (1.2-13.8)	0.0-56.0	99.7 (99.6-99.8)	18.2 (16.2-20.4)	< 0.0001	0.146	
**Europe**		1	5440	5.1 (3.4-7.2)	NA	NA	NA	NA	NA	
**South-East Asia**		9	62197	6.2 (2.3-12.0)	0.0-37.0	98.0 (97.3-98.5)	7.0 (6.1-8.2)	< 0.0001	0.094	
**Western Pacific**		5	14741	28.7 (15.4-46.0)	0.0-117.1	96.7 (94.4-98.0)	5.5 (4.2-7.1)	< 0.0001	0.612	
**Africa**	**A>E**	4	15196	7.2 (0.9-20.0)	0.0-112.2	97.0 (94.7-98.3)	5.8 (4.3-7.7)	< 0.0001	0.944	< 0.0001
**The Americas**		1	5996	41.9 (36.9-47.1)	NA	NA	NA	NA	NA	
**Eastern Mediterranean**		8	269417	7.5 (1.6-17.4)	0.0-64.4	99.7 (99.7-99.8)	19.3 (17.8-21.1)	< 0.0001	0.084	
**Europe**		1	6035	1.2 (0.4-2.2)	NA	NA	NA	NA	NA	
**South-East Asia**		14	465296	2.9 (1.4-4.9)	0.0-14.4	98.7 (98.4-98.9)	8.7 (7.9-9.6)	< 0.0001	0.128	
**Western Pacific**		3	11933	21.0 (7.2-41.8)	0.0-632.6	97.8 (95.7-98.8)	6.7 (4.8-9.2)	< 0.0001	0.219	
**Africa**	**E>E**	16	36250	24.4 (15.8-34.6)	0.3-82.2	97.0 (96.1-97.7)	5.7 (5.0-6.5)	< 0.0001	0.411	< 0.0001
**The Americas**		5	9186	16.4 (2.9-39.8)	0.0-160.0	97.7 (96.3-98.5)	6.6 (5.2-8.3)	< 0.0001	0.921	

**Eastern Mediterranean**		1	48930	2.9 (2.4-3.4)	NA	NA	NA	NA	NA	
**Europe**		1	684	74.6 (56.0-95.5)	NA	NA	NA	NA	NA	
**South-East Asia**		8	98779	9.2 (3.5-17.4)	0.0-52.3	99.0 (98.6-99.2)	9.8 (8.5-11.2)	< 0.0001	0.024	
**Western Pacific**		15	103080	27.2 (16.2-40.9)	0.0-105.9	99.2 (99.1-99.4)	11.5 (10.5-12.5)	< 0.0001	0.895	
Areas										
**Rural**	**WHF**	4	9653	31.1 (20.8-43.5)	0.4-105.1	89.8 (76.8-95.5)	3.1 (2.1-4.7)	< 0.0001	0.148	0.090
**Urban**		10	34466	15.6 (8.4-24.9)	0.0-61.7	96.9 (95.6-97.8)	5.7 (4.8-6.7)	< 0.0001	0.616	
**Both**		13	82909	24.3 (13.2-38.5)	0.0-102.7	99.1 (98.9-99.3)	10.6 (9.6-11.7)	< 0.0001	0.467	
**Rural**	**WHO**	3	12269	8.3 (1.2-21.5)	0.0-518.2	97.8 (96.4-98.7)	6.8 (5.2-8.8)	< 0.0001	0.509	0.415
**Urban**		15	259373	11.8 (6.1-19.4)	0.0-57.4	99.3 (99.2-99.4)	11.9 (11.0-13.0)	< 0.0001	0.0005	
**Both**		8	49948	16.0 (5.0-33.1)	0.0-107.6	99.3 (99.1-99.4)	11.9 (10.6-13.4)	< 0.0001	0.131	
**Rural**	**A>E**	6	291357	4.5 (0.9-10.9)	0.0-41.6	99.2 (99.0-99.4)	11.3 (10.0-12.8)	< 0.0001	0.266	0.635
**Urban**		18	312681	8.1 (3.6-14.3)	0.0-51.4	99.5 (99.4-99.6)	14.2 (13.2-15.2)	< 0.0001	0.008	
**Both**		5	49780	5.6 (0.1-18.8)	0.0-100.2	99.5 (99.3-99.6)	13.8 (12.0-15.9)	< 0.0001	0.349	
**Rural**	**E>E**	6	24360	14.5 (5.5-27.7)	0.0-83.3	98.1 (97.2-98.7)	7.2 (6.0-8.8)	< 0.0001	0.415	0.741
**Urban**		14	53138	17.5 (12.4-23.6)	1.9-47.8	95.2 (93.3-96.5)	4.5 (3.9-5.4)	< 0.0001	0.018	
**Both**		21	16224	20.6 (10.8-23.6)	0.0-112.3	99.5 (99.5-99.6)	14.9 (14.0-15.8)	< 0.0001	0.265	
Settings										
**Community-based**	**WHF**	7	44190	30.7 (19.8-44.0)	2.6-5.3	94.2 (90.4-96.5)	4.1 (3.2-5.3)	< 0.0001	0.096	0.302
**School-based**		24	103079	24.6 (17.5-32.8)	0.7-79.4	98.3 (98.0-98.6)	7.8 (7.1-8.5)	< 0.0001	0.037	
**Both**		1	1450	33.8 (25.1-43.7)	NA	NA	NA	NA	NA	
**Community-based**	**WHO**	1	489	22.5 (11.0-37.8)	NA	NA	NA	NA	NA	0.06
**School-based**		28	377514	11.0 (7.0-16.0)	0.0-49.6	99.3 (99.2-99.3)	11.6 (10.9-12.3)	< 0.0001	< 0.0001	
**Both**		0	NA	NA	NA	NA	NA	NA	NA	
**Community-based**	**A>E**	4	62666	6.0 (3.2-9.7)	0.0-30.6	94.0 (87.9-97.1)	4.1 (2.9-5.8)	< 0.0001	0.216	0.732
**School-based**		26	593195	6.8 (3.9-10.4)	0.0-32.2	99.4 (99.3-99.5)	13.0 (12.2-13.7)	< 0.0001	0.002	
**Both**		0	NA	NA	NA	NA	NA	NA	NA	
**Community-based**	**E>E**	8	54439	19.1 (4.7-42.5)	0.0-146.6	99.3 (99.2-99.5)	12.2 (10.9-13.8)	< 0.0001	0.276	0.846
**School-based**		36	235097	22.2 (16.3-29.1)	0.0-79.0	99.0 (98.9-99.1)	10.1 (9.5-10.7)	< 0.0001	< 0.0001	
**Both**		2	7373	12.1 (0.0-64.1)	NA	99.0 (98.1-99.5)	10.1	< 0.0001	NA	

NA: not applicable; WHF: World Heart Federation; WHO: World Heart Organization; UNSD: United Nations Statistical Divisions; A>E = Auscultation only for screening followed with echography confirmation; E>E: Echography +/- auscultation for screening followed with echography for confirmation; NA = not applicable

Supplementary Figure 1. Funnel plot for publication bias for studies using the WHO diagnostic criteria

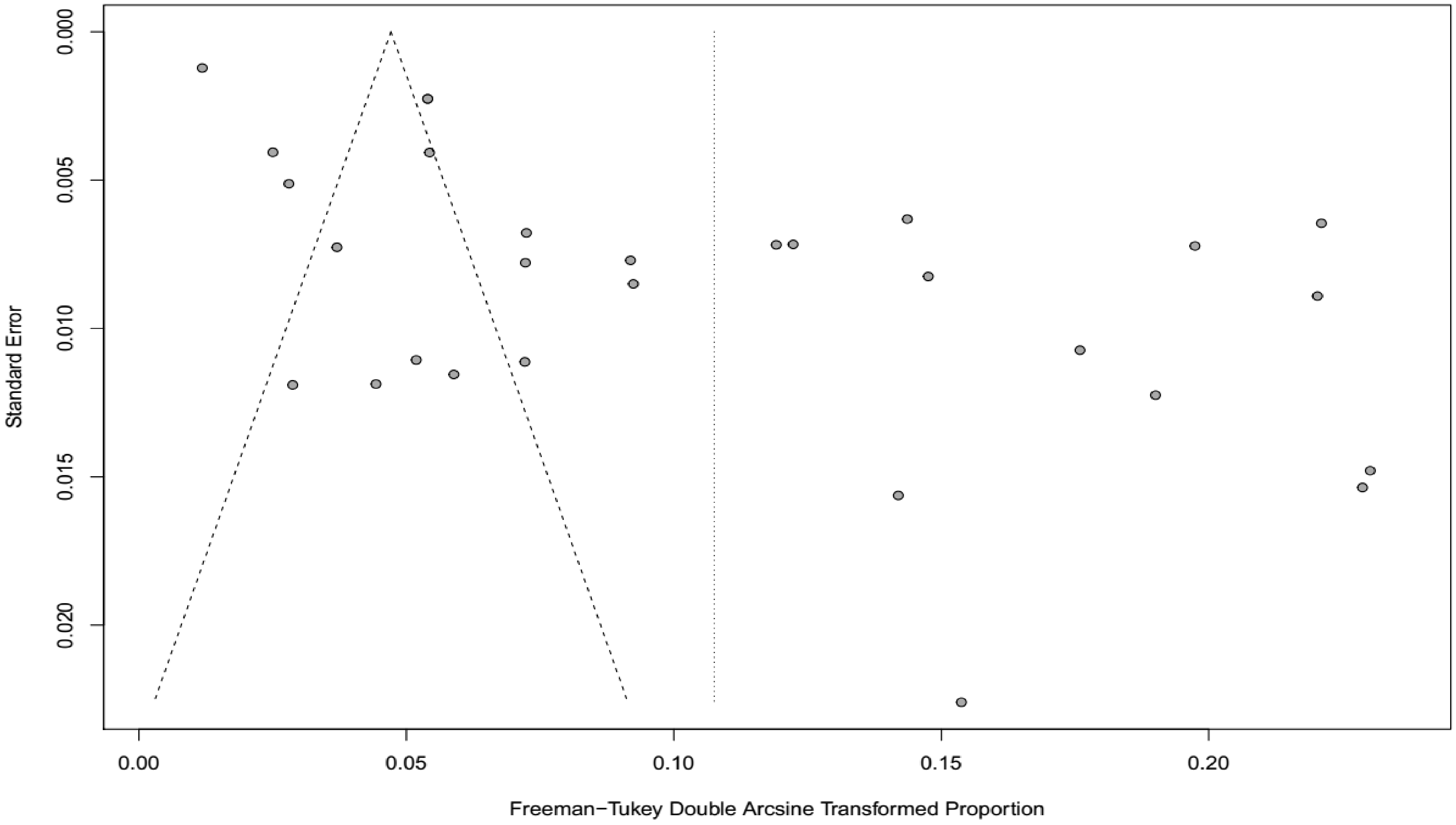

Supplementary Figure 2. Funnel plot for publication bias for studies using the WHF diagnostic criteria

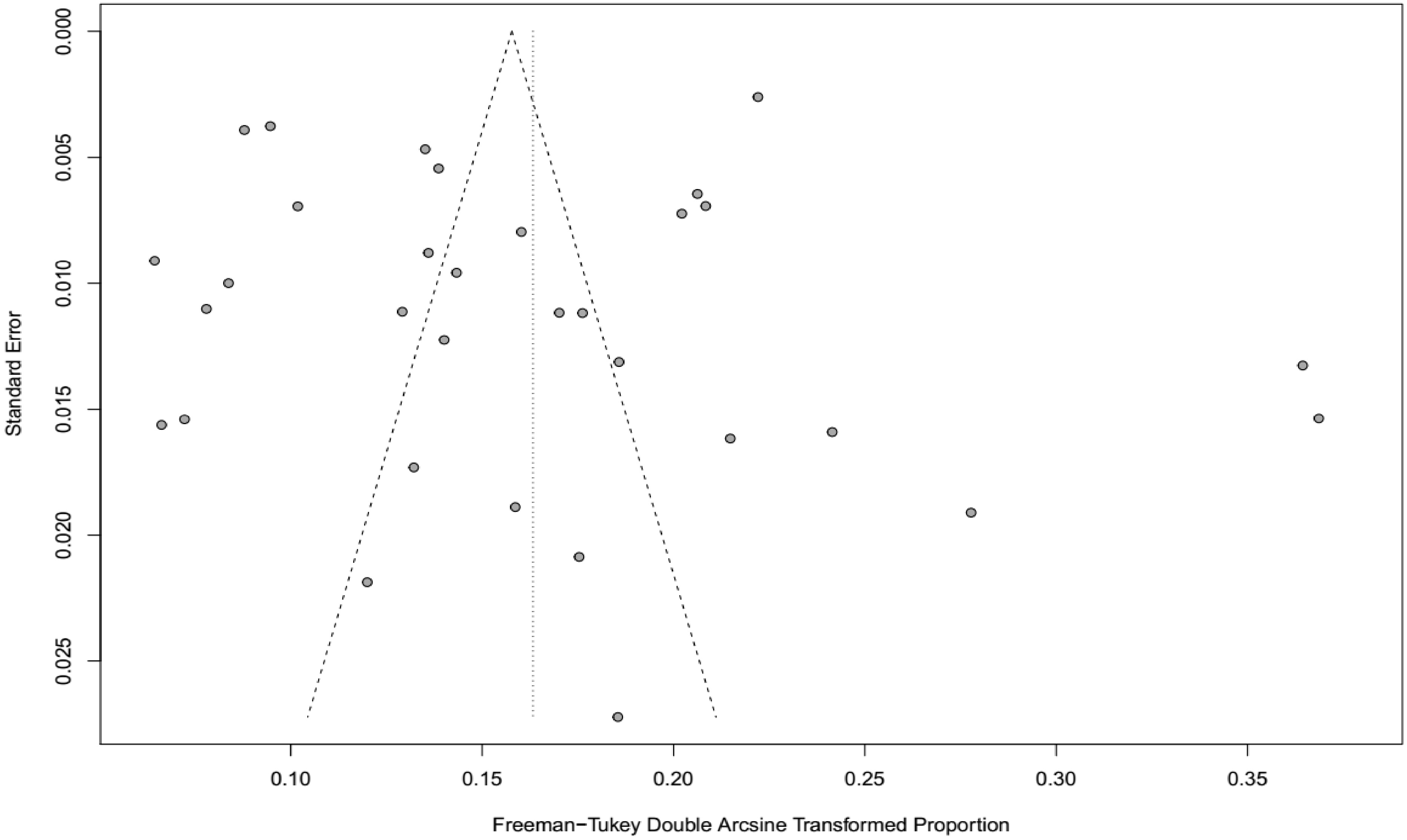

Supplementary Figure 3. Funnel plot for publication bias for studies using diagnostic criteria other than WHF and WHO

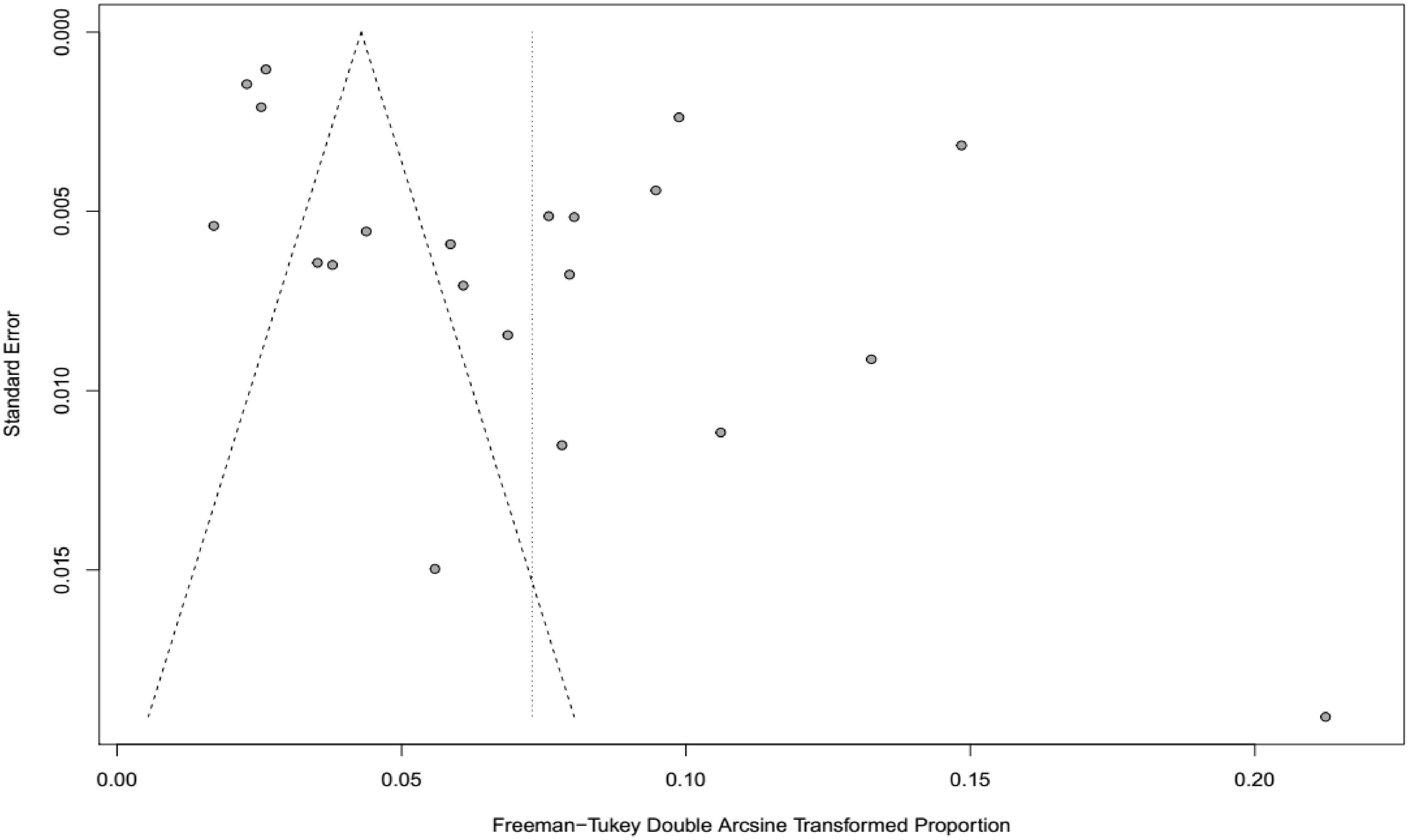

Supplementary Figure 4. Funnel plot for publication bias for studies using an auscultation-echocardiographic screening protocol

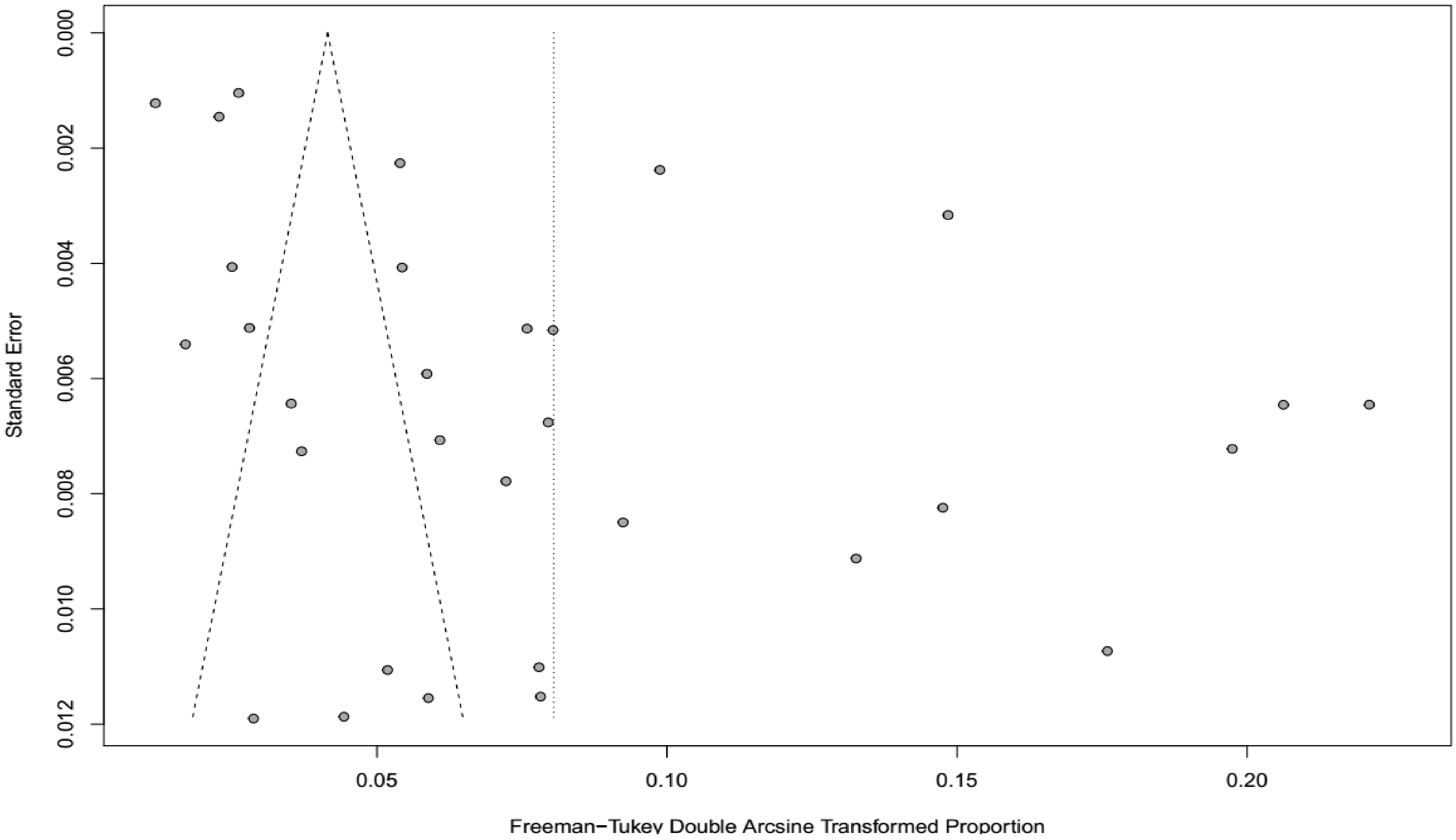

Supplementary Figure 5. Funnel plot for publication bias for studies using an echocardiography-echocardiography screening protocol

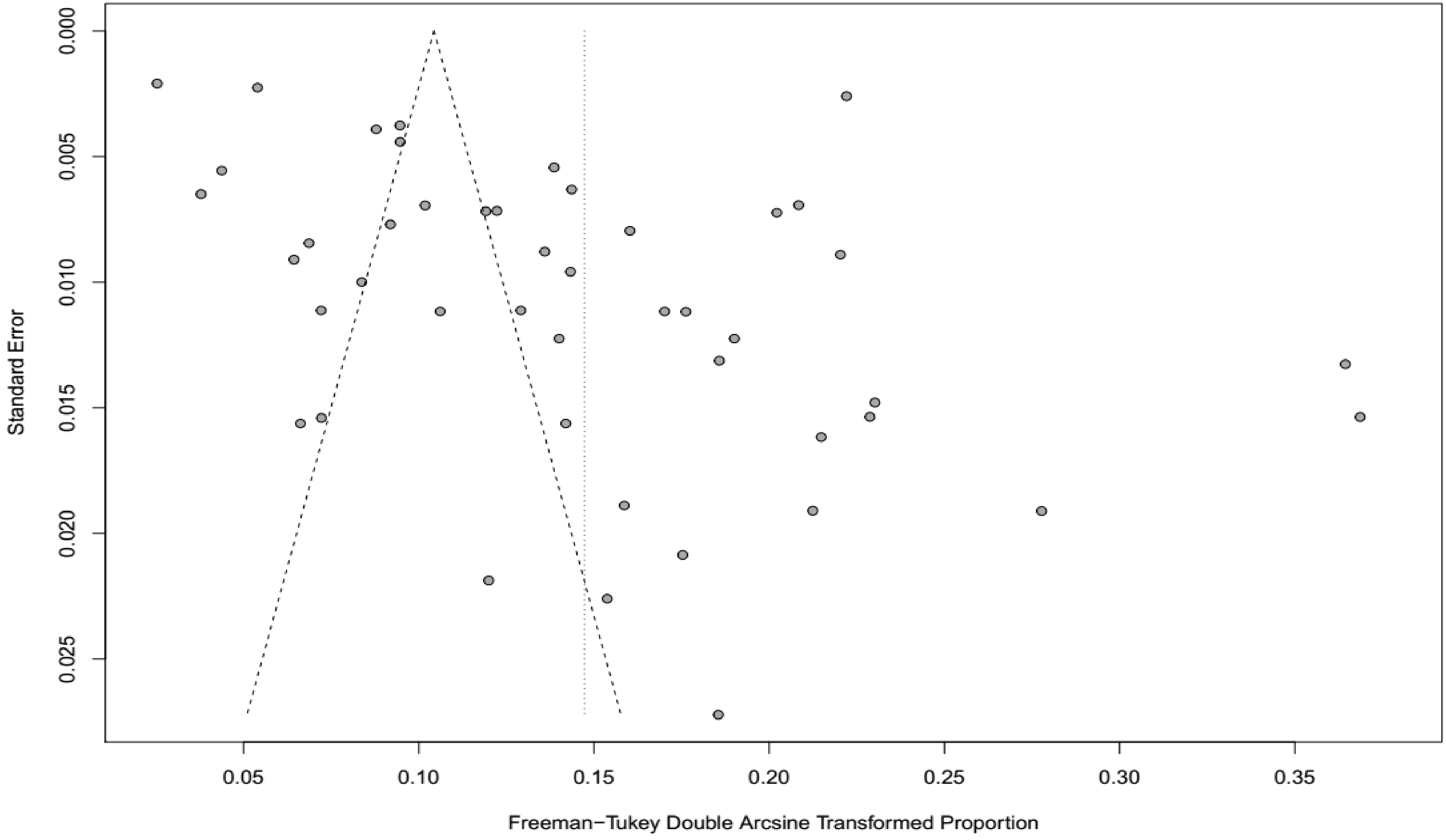

Supplementary Figure 6. Funnel plot for publication bias for studies using only one step echocardiography screening protocol

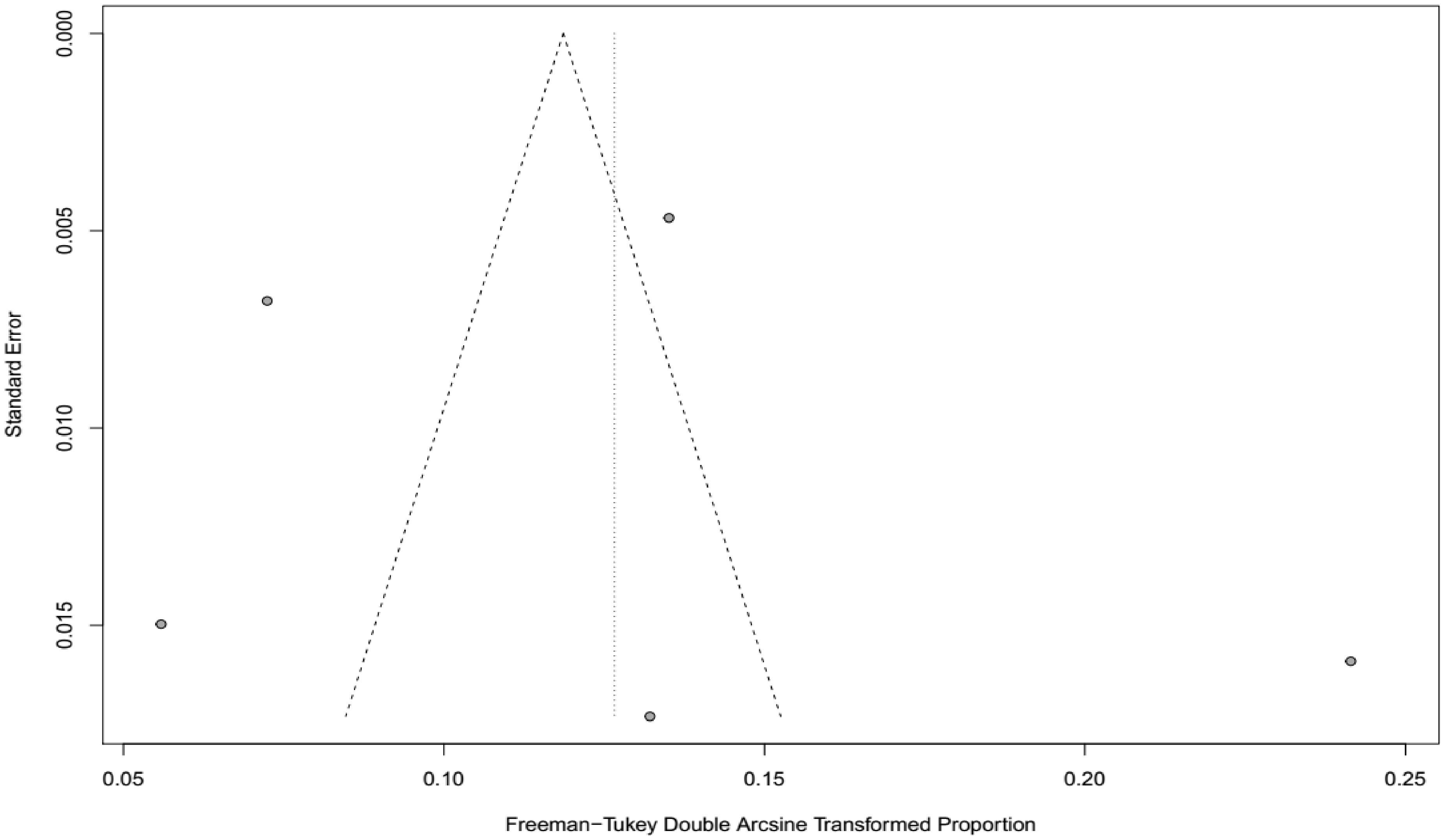

Supplement: Supplementary file 1 — Appendix [file 41598_2019_53540_MOESM1_ESM.pdf]
